# Supplementary material for: Stereo-random oligonucleotides enable efficient recruitment of ADAR in vitro and in vivo
Source: Nat Commun. 2025 Oct 3;16:8849. doi: 10.1038/s41467-025-64434-7 (PMC12494997; doi:10.1038/s41467-025-64434-7)
Supplement: Supplementary file 1 — Supplementary Information [file 41467_2025_64434_MOESM1_ESM.pdf]

Supplementary Information

Inventory of Supplementary Information:

- Supplementary Figures 1 - 8
- Supplementary Methods
- Supplementary Table 1
- Supplementary Figures 9 + 10
- Supplementary Sequences 1 - 6
- References

Supplementary Figures

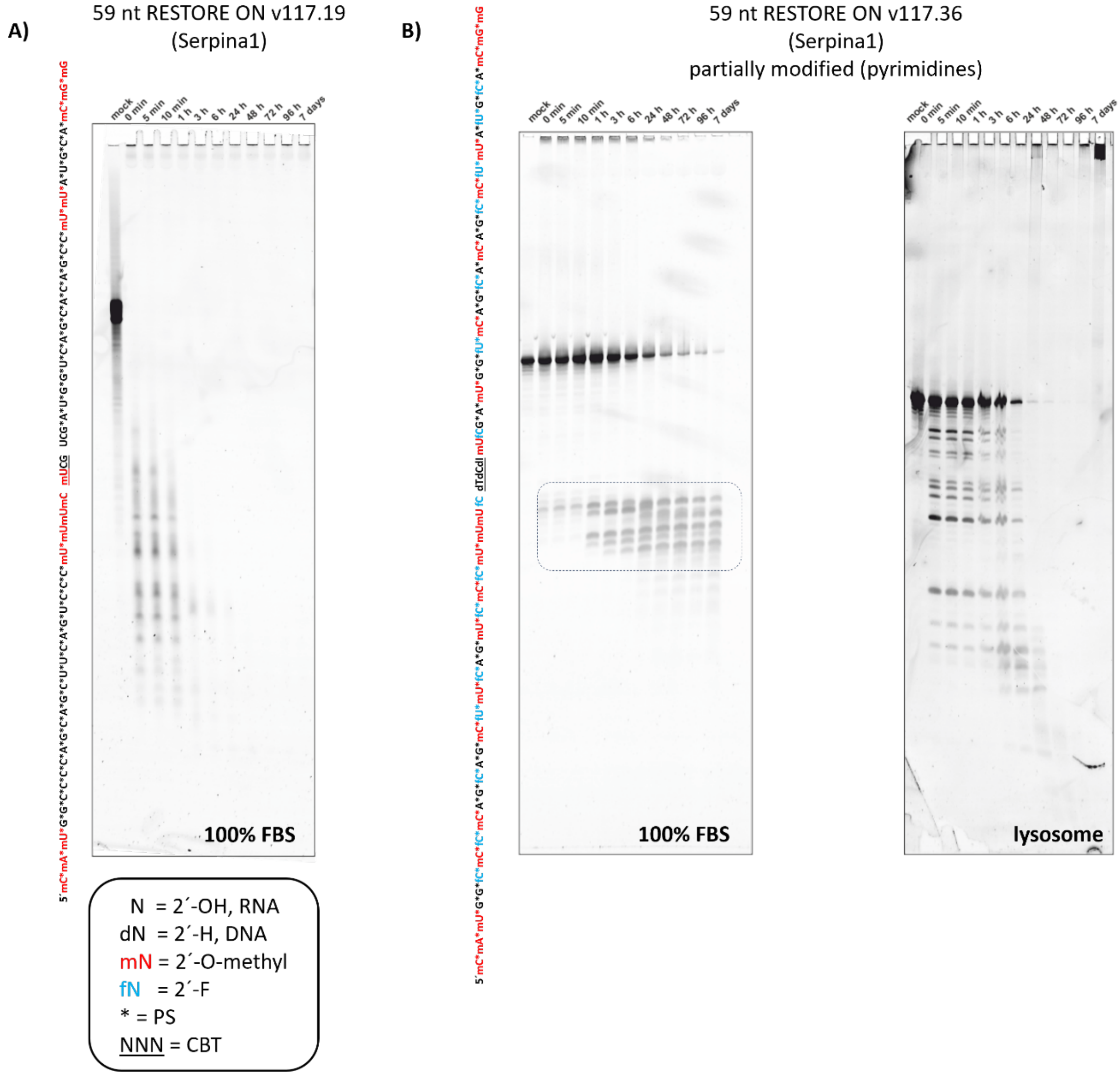

Supplementary Figure 1 | Degradation of partially modified RESTORE 2.0 ONs. Shown are the full urea PAGE gels of a minimally (v117.19) and a partially modified (v117.26) RESTORE 2.0 ON of 59 nt length, modification pattern as indicated in the boxed legend. **A)** The degradation pattern of v117.26 in 100% FBS fits to a low number of major breakpoints (dotted box) inside the DNA-modified CBT, fitting well to the presence of DNase I in FBS. **B)** In contrast, the degradation pattern in lysosome is more complex, including breakpoints outside the CBT.

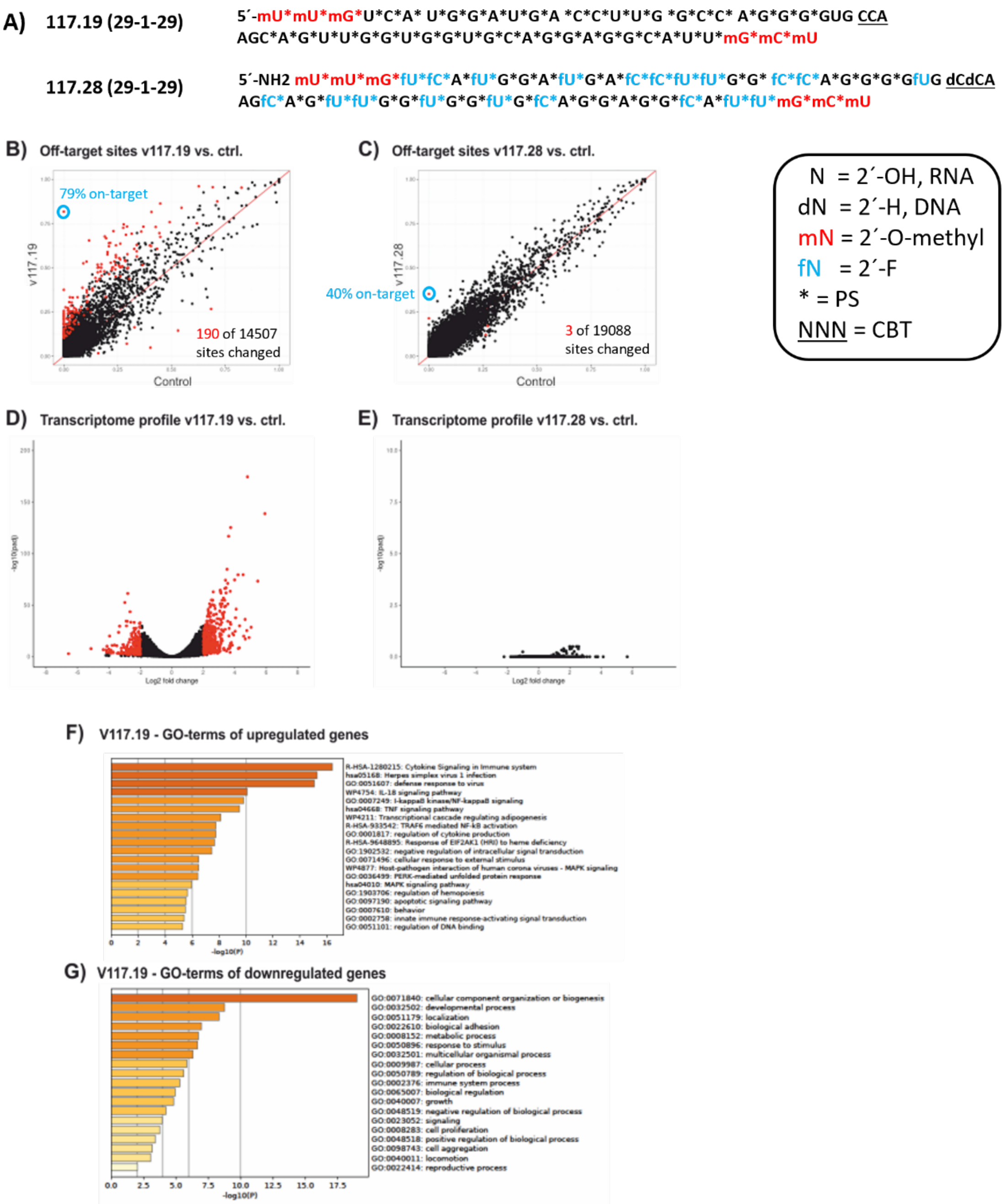

Supplementary Figure 2 | Global off-target analysis of RESTORE ONs by next generation sequencing (NGS) RNA seq. A minimally modified 59 nt GAPDH ORF-targeting ON (v117.19) was compared to a partially modified 59 nt GAPDH ORF-targeting ON (v117.28) with 2´-F modification at pyrimidine bases, after transfection into primary human astrocytes (NHA). **A)** Sequence and modification of the compared ONs. **B)** For the unmodified ON, 190 significantly differently edited sites were discovered. Overall, the global editing seems shifted towards higher editing levels. **C)** For the partially modified ON, off-target editing was negligible. **D)** The unmodified ON led to significant changes in gene expression. **E)** The partially modified ON did not lead to changes in gene expression. **F)**, **G)** Gene ontology analysis revealed an upregulation of the interferon-stimulated gene (ISG) response and a downregulation of proliferation in response to ON v117.19. This effect is not seen after further chemical modification of the ON (v117.28). The editing levels of edited sites were quantified with ≥50 reads coverage (combined coverage of both

replicates) and Fisher's exact tests followed by Benjamini–Hochberg's multiple test correction (adjusted $P < 0.01$) and were performed to identify significantly differently edited sites across the samples. Differential expressed genes (DEGs) were identified using DESeq2 with default settings (FDR cut-off = 1e-3), panel D and E. GOrilla was used to identify enriched GO terms in up-/downregulated genes (FDR q-value < 1e-5, enrichment > 1.5), panel F and G. For further detail, see Supplementary Methods.

A) MECP2 W104X

guide RNA	PS positions	15% denaturing urea PAGE (100% FBS)	t_{50} (100% FBS)	Editing yield [%]
V120.17	a, j	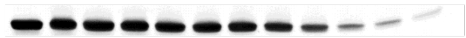	6 h	49,7% ± 1,8 (n = 3)
V120.23	a, d, e, f, j	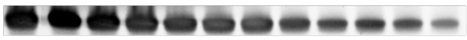	84 h	50,7% ± 1,8 (n = 3)
V120.24	a - j	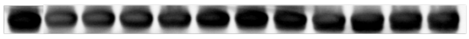	>7 days	29,9% ± 3,3 (n = 3)

mock, 0 min, 5 min, 10 min, 1 h, 3 h, 6 h, 24 h, 48 h, 72 h, 96 h, 7 days

B) STAT1 Y701

guide RNA	PS positions	15% denaturing urea PAGE (100% FBS)	t_{50} (100% FBS)	Editing yield [%]
V117.28	a, j	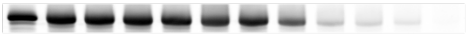	30 h	40,5% ± 1,3 (n = 3)
V117.29	a, d, e, f, j	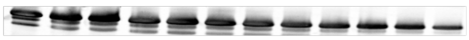	6 days	50,5% ± 4,1 (n = 3)

mock, 0 min, 5 min, 10 min, 1 h, 3 h, 6 h, 24 h, 48 h, 72 h, 96 h, 7 days

Supplementary Figure 3 | Transfer of optimal PS/PO-linkage positioning at the CBT for additional targets. Shown are stability assays in 100% FBS and editing yields in HeLa cells for ONs targeting **A)** the disease-causing murine MECP2 W104X (5’-UAG) site and **B)** the regulatory phosphotyrosine Y701 in human STAT1 (5’-UAU). Uncropped gel images are supplied in Supplementary raw data 11 + 12.

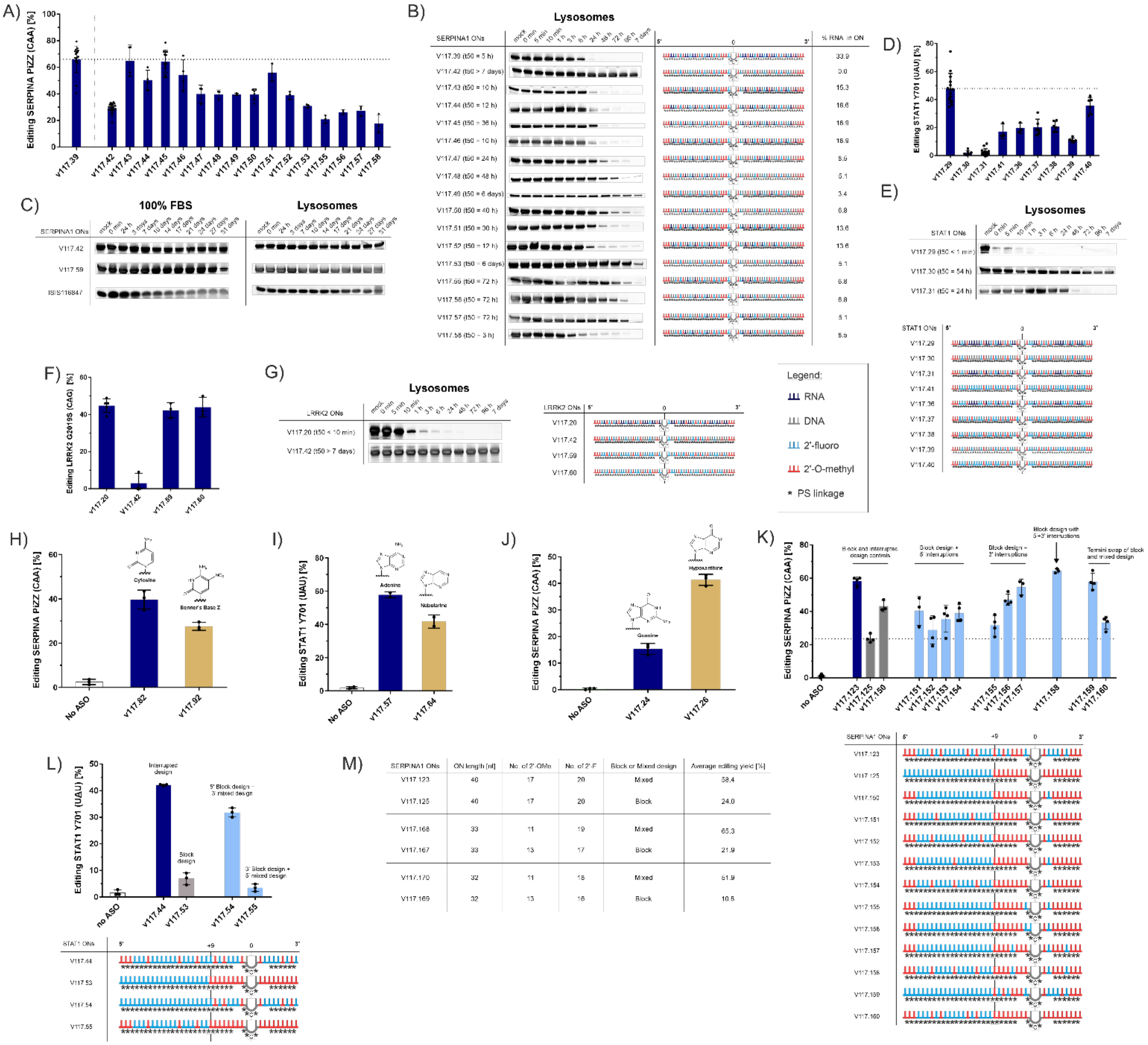

Supplementary Figure 4 | Additional information on the full modification of RESTORE v2.0 ONs. All editing yields were obtained in HeLa cells, or in a HeLa cell model expressing the SERPINA1 E342K cDNA. **A)** Editing yields after gradual addition of DNA to unmodified purine bases in partially modified ONs targeting the human SERPINA1 E342K (PiZZ) site. **B)** Lysosomal stability assay and modification scheme of ONs from panel A). **C)** Long-term (31 days) stability assay of fully modified ONs targeting the human SERPINA1 E342K site in 100% FBS or lysosomal fluid. **D)** Editing yields of partially modified ONs targeting the human STAT1 Y701 site after partial or full conversion of unmodified purine bases to DNA and/or 2'-F. **E)** Lysosomal stability assay and modification scheme of ONs from panel D). **F)** Editing yields of ONs targeting the human LRRK2 G2019S site before and after full modification with DNA and/or 2'-F. **G)** Lysosomal stability assay and modification scheme of ONs from panel F). **H)** Side-by-side editing yield comparison of ONs with (v117.92) or without (v117.82) the Benner's Base Z[1] as the orphan base targeting the human SERPINA1 E342K site. **I)** Side-by-side editing yield comparison of ONs with (v117.64)

or without (v117.57) a nebularine base placed directly 3'-adjacent of the orphan cytosine[2] targeting the human STAT1 Y701>C site. **J)** Side-by-side comparison of ONs with (v117.26) or without (v117.24) an inosine/hypoxanthine placed directly 3'-adjacent of the orphan cytosine targeting the human SERPINA1 E342K site. **K)** Editing yields and modification schemes of intermediate Block and Mixed ON designs targeting the human SERPINA1 E342K site. **L)** Editing yields and modification schemes of intermediate Block and Mixed ON designs targeting the human STAT1 Y701>C site. **M)** Side-by-side comparison of absolute ribose modification content and editing yields of Block and Mixed ON designs targeting the SERPINA1 E342K site from Figure 4F.

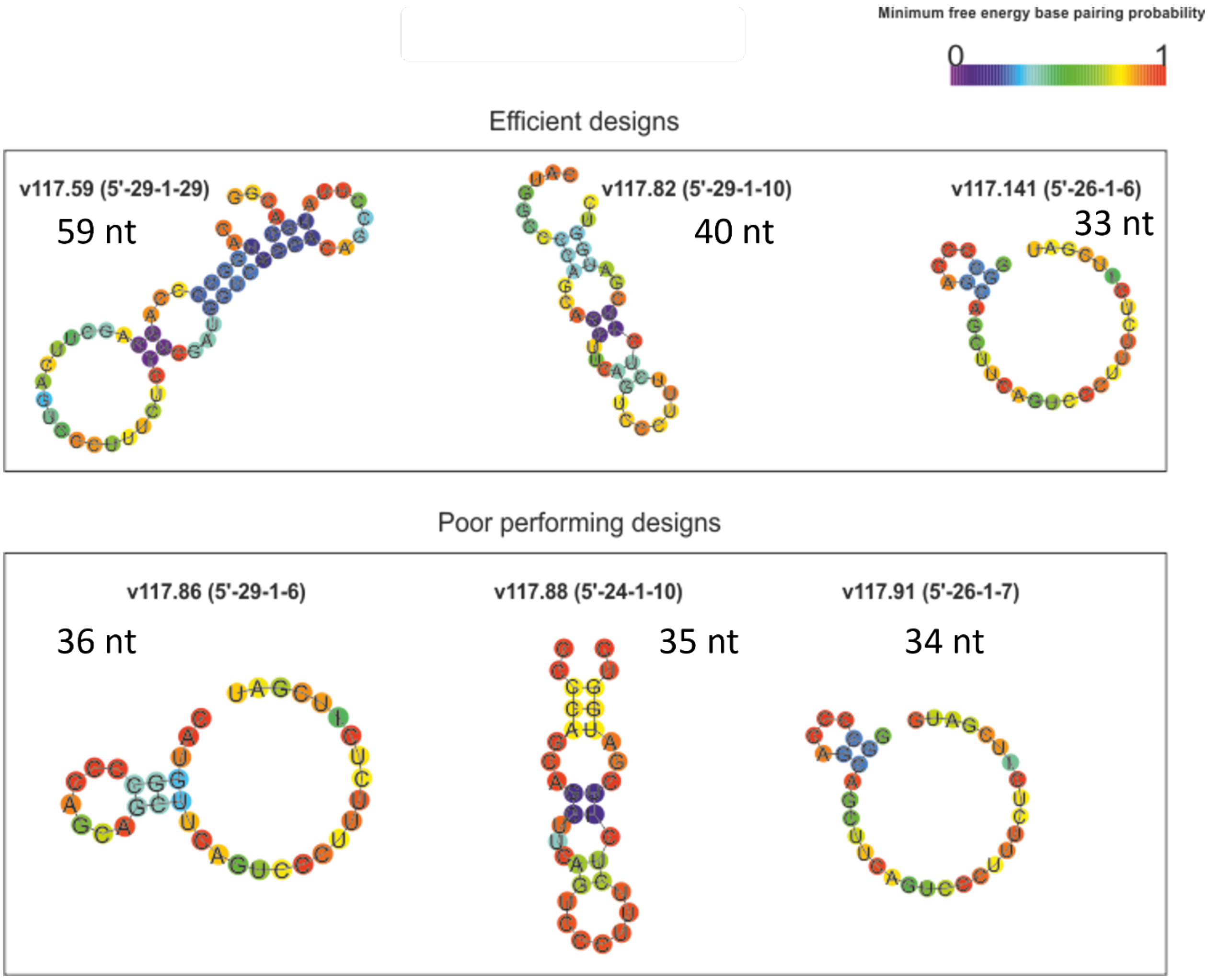

Supplementary Figure 5 | Analysis of the secondary RNA structure of selected RESTORE 2.0 ON designs of different length. Stable secondary structure / self-folding of RESTORE ONs could influence their editing performance. However, we do not see notable differences in the structures of RESTORE 2.0 ON targeting the Serpina1 PiZ mutation when comparing efficient designs (good editing performance) with poor designs (low editing performance) of various lengths. Shown are minimum free energy (MFE) folded RNAs, simulated with the RNAFold web server of the University of Vienna[3].

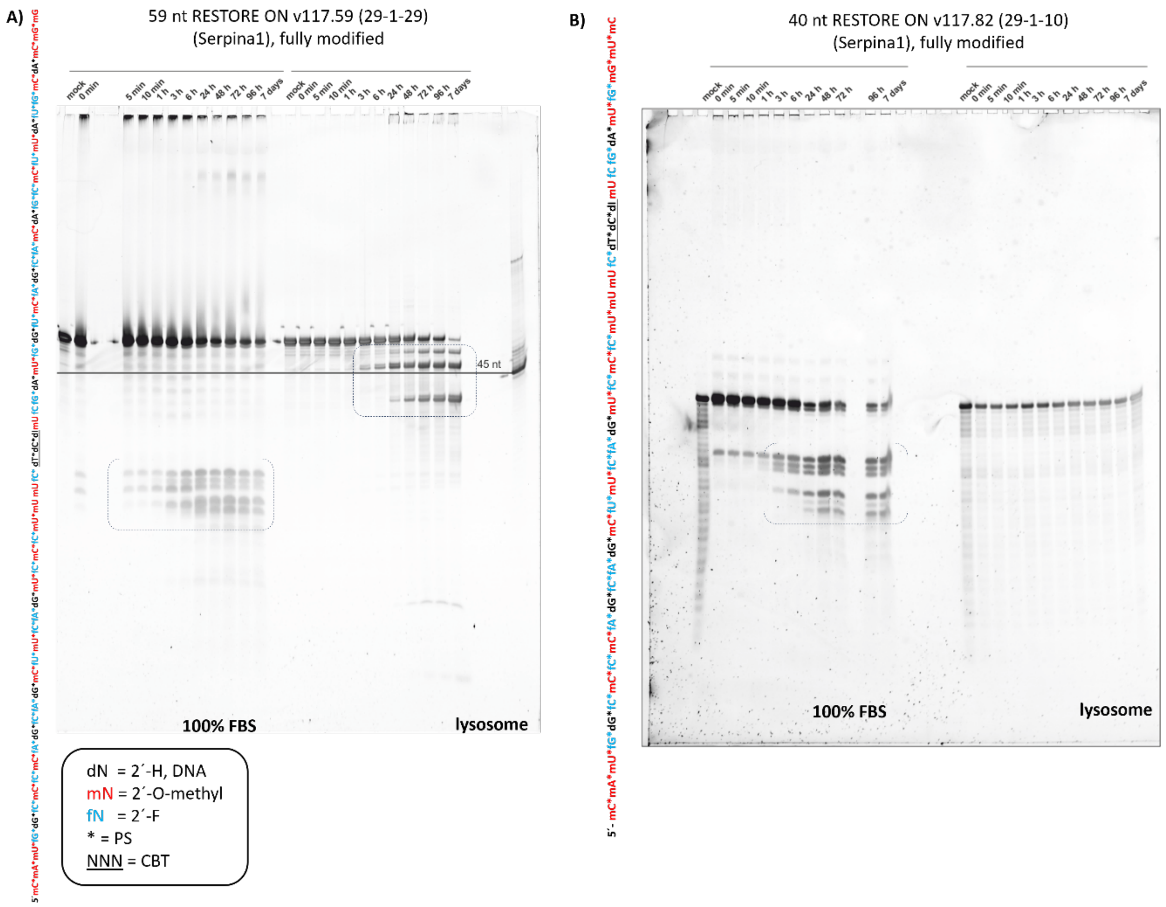

Supplementary Figure 6 | Degradation of fully modified RESTORE 2.0 ONs. Shown are the full urea PAGE gels of a long (59 nt, v117.59) and short (40 nt, v117.82) fully modified RESTORE ON, modification patterns as indicated in the boxed legend. **A)** degradation pattern of v117.59 in 100% FBS and lysosome. In FBS, the major breakpoint (dotted box) is the CBT; in lysosome, the major break points (dotted box) seem to be located in the (3´) flank of the oligonucleotide. **B)** The 40 nt ON v117.82 does not contain these labile sites and does not give a clear degradation pattern in lysosome. However, in FBS the major degradation (dotted box) happens mainly in the CBT region. The degradation patterns fit well to the presence of DNase I and RNase A in FBS and RNase T1/2 in lysosome.

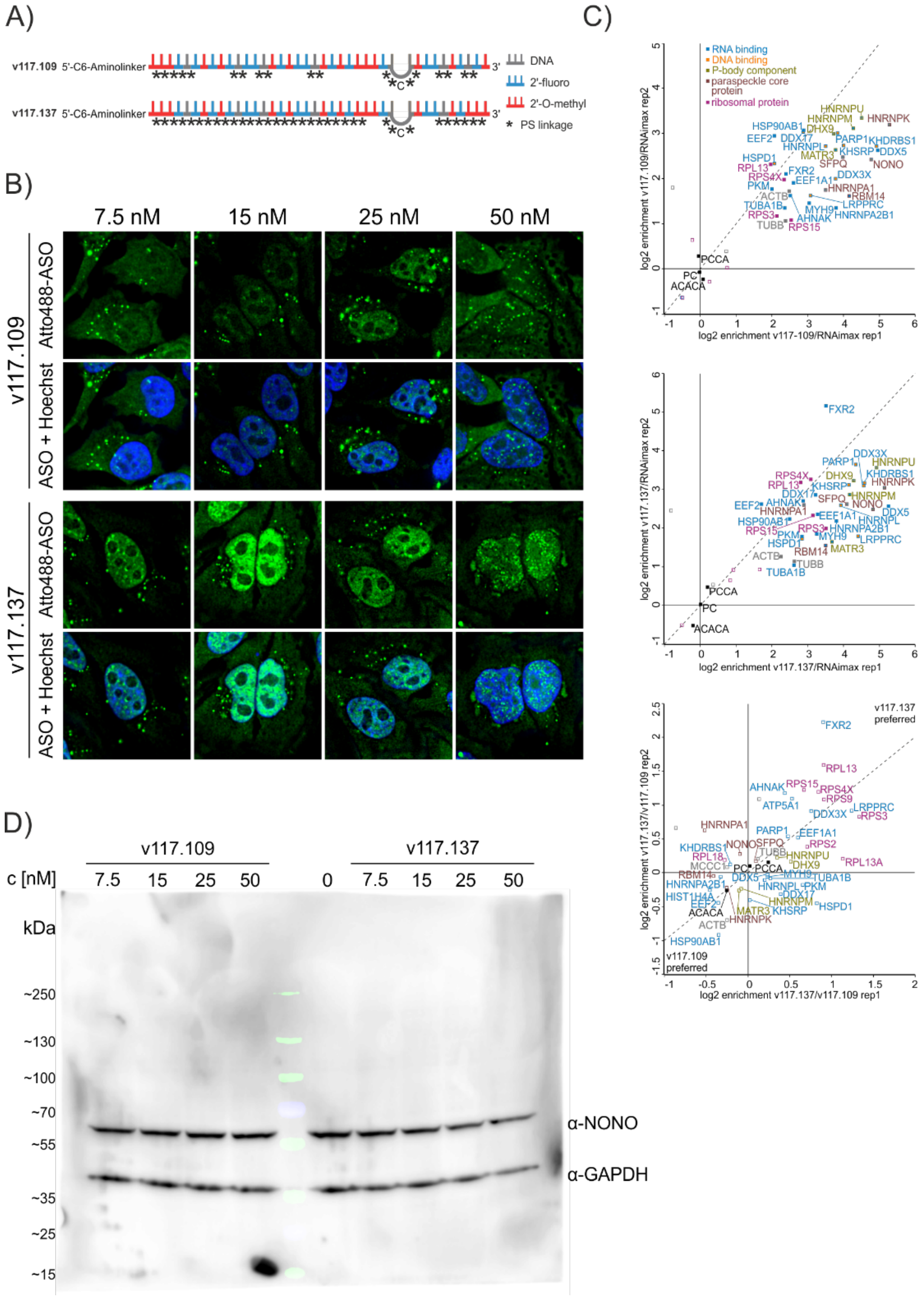

Supplementary Figure 7 |Intracellular behavior of RESTORE 2.0 ON. A) Two 40 nt RESTORE 2.0 ON (5'-29-1-10) targeting the PiZ mutation in SERPINA1 are compared, one with high (v117.137), one with low PS content (v117.109). **B)** Intracellular ON localization. The PS-rich ON seems more stable and slightly more localized to the nucleus compared to the PS-low variant. Both ONs do not form large nucleolar aggregates, or nuclear filaments as observed for certain gapmers in the literature[4]. Toxic gapmers often tend to delocalize paraspeckle proteins into nucleoli. n = 1. **C)** Binding interactome of the two ONs. Both bind to classical, nuclear paraspeckle proteins like NONO and SFPQ or P-body components. In their direct comparison, the PS-rich variant gives higher enrichment factors, indicating a stronger protein binding. The interactome was obtained with the isASO-ID protocol[5]. All ratios were normalized to the geometric

mean of three carboxylases highlighted in black. RNA binding = blue, DNA binding = orange, P-body component = olive green, paraspeckle core protein = dark red, ribosomal protein = purple; rep = replicate. n = 2 independent experiments. **D)** We do not have any indication that RESTORE 2.0 ONs´ binding to NONO leads to its degradation, as seen for some 2´-F containing gapmers in the literature[6]. n = 1.

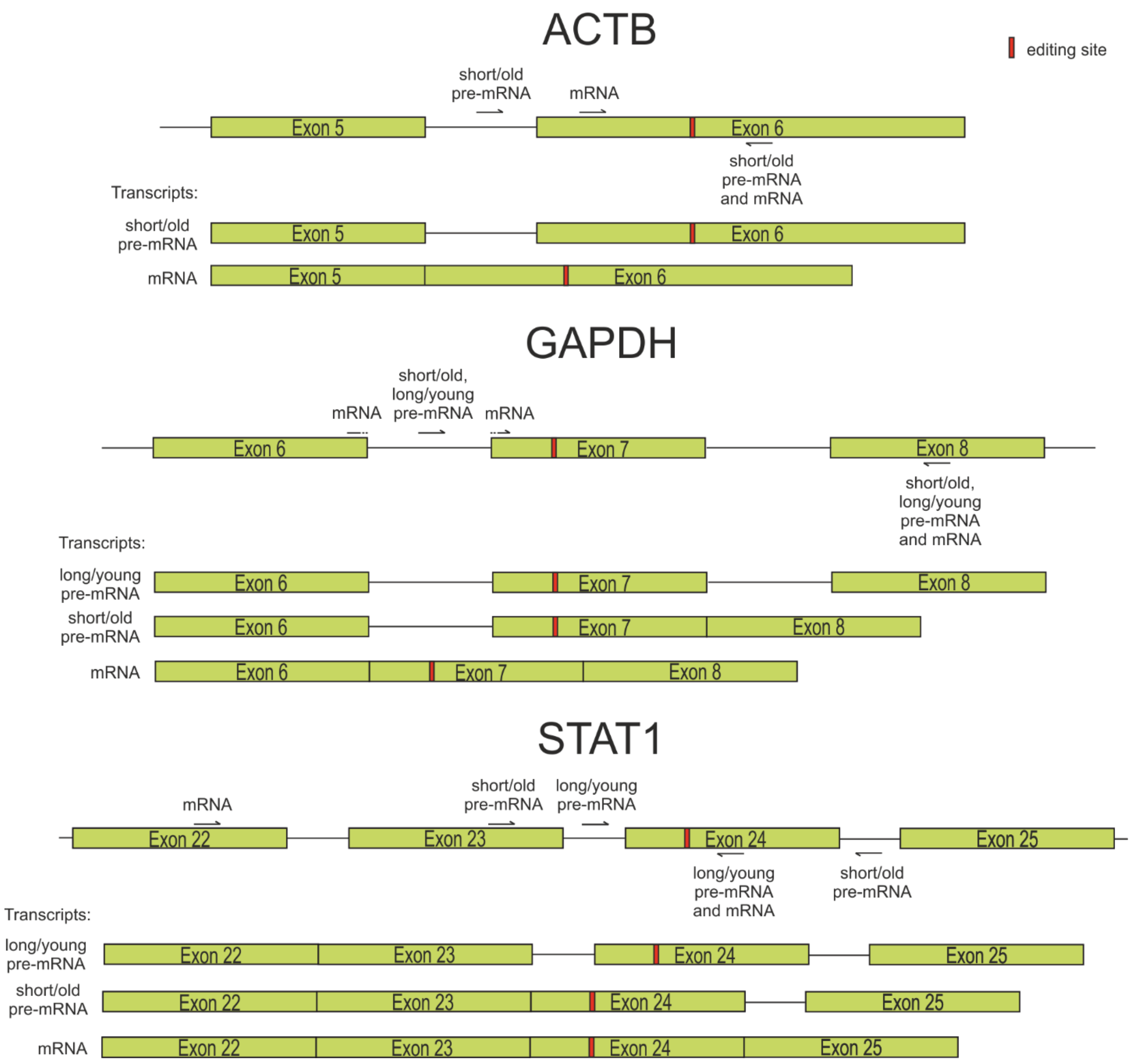

Supplementary Figure 8 |Assessment of editing throughout the splicing process. Illustration showing the different, assessed transcripts isoforms along the splicing process of ACTB, GAPDH and STAT1 around their respective editing sites (marked as a red vertical line). For each gene the non-spliced pre-mRNA is illustrated with exons (green boxes) and introns (black lines), respectively, and with the respective primer pairs (arrows) that have been used to assess the editing yields of differently spliced isoforms via RT-PCR. Furthermore, all differently spliced pre-mRNA isoforms (transcripts), which have been assessed, are shown. RT-PCR products of different transcript isoforms were separated depending on their different lengths by agarose gel electrophoresis prior to Sanger sequencing. The latter was used to confirm the transcript isoform and to determine the editing yield in that isoform.

Supplementary Methods

Editing procedure with ONs targeting the human LRRK2 G2019S site. $5x10^4$ HeLa cells/well (cat. no. ATCC CCL-2) in 500 µl DMEM plus 10% FBS were seeded into a 24-well plate. After 24h, cells were forward transfected with a plasmid containing the human LRRK2 G2019S cDNA (pTS1560, a generous gift from the Gloeckner Lab (DZNE)). For this, 300 ng plasmid and 0.9 µl FuGENE6® (Promega) were each diluted to a total volume of 50 µl with OptiMEM in separate tubes. After 5 min, both solutions were mixed and incubated for an additional 20 min. Medium was changed on the cells prior to dropwise addition of the ready plasmid transfection mix to each well. 24 h after plasmid transfection, ONs were transfected as described for the immortalized cell lines. 24h after ON transfection, cells were harvested for RNA isolation and sequencing. Results are reported in Supplementary Fig. 2F. The LRRK2 plasmid map is depicted in Supplementary Note 1.

Editing procedure with ONs targeting the murine MECP2 W104X site. HeLa cells (cat. no. ATCC CCL-2) stably expressing the MECP2 W104X mutation were generated as previously described for the HeLa cells containing genomically integrated SERPINA1 E342K (ref.[7]), with minor differences. The wildtype MECP2 cDNA was isolated by reverse transcription of total RNA from mouse embryonic fibroblasts. The W104X mutation was inserted into the cDNA via PCR. Before insertion of the MECP2 cDNA, eGFP cDNA was inserted into a PB-CA vector using NheI and XhoI restriction. Afterwards, the MECP2 W104X-mutated cDNA was inserted into the PB-CA-eGFP vector under control of a CMV promotor using XbaI and BamHI restriction (plasmid name: pTS1088). Genomic integration via the piggyBac transposon system and puromycin selection of positive cells was performed as described[7]. The further editing procedure with RESTORE v2.0 ONs was performed as described for the immortalized cell lines. Results are reported in Supplementary Fig. 1A. The MECP2 W104X-eGFP plasmid map is depicted in Supplementary Note 2.

Next-generation RNA sequencing of ONs in primary normal human astrocytes (NHA). NHAs were seeded and transfected with ONs and lysed as described in the manuscript. Overall, three settings were carried out, each with an independent duplicate: 1) empty lipofection ("ctrl." in plots), 2) transfection with minimally modified GAPDH ORF-targeting ON v117.19 and 3) transfection with partially modified GAPDH ORF-targeting ON v117.28. RNA was isolated 24 h after transfection with the RNeasy MinElute Kit according to manufacturer's protocol, treated with DNase I, incubated with an RNA strand reverse complementary to the respective ON and heated to 95 °C for 3 min and purified again with the RNeasy MinElute Kit. Purified RNA was delivered to CeGaT (Germany) for poly(A)$^+$ mRNA sequencing. The library was prepared from 100 ng RNA with the TruSeq Stranded mRNA Library Prep Kit (Illumina, USA) and sequenced with a NovaSeq 6000 (50M reads, 2 × 100 bp paired end, Illumina, USA). Subsequent downstream bioinformatical analysis was performed as previously described[7, 8]. Briefly, BWA (version 0.7.10)[9] was used to align the reads to a combination of the reference genome sequences and exonic sequences surrounding known splicing junctions from known gene models. Each of the paired-end reads was mapped separately using the commands "bwa aln fastqfile" and "bwa samse -n4". The length of the splicing junction was chosen to be slightly shorter than the RNA-seq reads to prevent redundant alignment (i.e., 95 bp for reads of 100 bp length). The reference genomes used were hg19 and the gene models were obtained through the UCSC Genome Browser for Gencode, RefSeq, Ensembl, and UCSC Genes. Only uniquely mapped reads with mapping quality $q > 10$ were used. Picard[v] was used to remove clonal reads (PCR duplicates) mapped to the same location. Of these identical reads, only the read with the highest mapping quality was kept for downstream analysis. Unique and nonduplicate reads were subjected to local realignment and base score recalibration using the IndelRealigner and

TableRecalibration from the Genome Analysis Toolkit (GATK, version 3.6)[10]. The above steps were applied separately to each of the RNA-seq samples. The UnifiedGenotyper from GATK[10] was used to call variants from the mapped RNA-seq reads. In contrast to typical variant calling, the variants were identified with relatively loose criteria by using the UnifiedGenotyper tool with options stand_call_conf 0, stand_emit_conf 0, and output mode EMIT_VARIANTS_ONLY. Variants from nonrepetitive and repetitive non-Alu regions were required to be supported by at least three reads containing mismatches between the reference genome sequences and RNA-seq. Supporting of one mismatch read was required for variants in Alu regions. This set of variant candidates was subject to several filtering steps to increase the accuracy of editing site calling. First, all known human SNPs present in dbSNP build 137 (except SNPs of molecular type "cDNA"; database version 135; http://www.ncbi.nlm.nih.gov/SNP/), the 1000 Genomes Project, and the University of Washington Exome Sequencing Project (http://evs.gs.washington.edu/EVS/) were removed. To remove false-positive RNA-seq variant calls due to technical artifacts, further filters were applied: a variant call quality of $Q > 20$ (refs. [11, 12]), variants were removed if they occurred in the first 6 bases of a read[9], variants in simple repeats were discarded[13], intronic variants that were within 4 bp of splice junctions were removed, and variants in homopolymers were discarded. Moreover, we removed reads mapped to highly similar regions of the transcriptome by BLAT[14]. Finally, variants were annotated using ANNOVAR (version 11122013)[15] based on gene models from Gencode, RefSeq, Ensembl and UCSC. The editing levels of edited sites were quantified with ≥50 reads coverage (combined coverage of both replicates) and Fisher's exact tests followed by Benjamini–Hochberg's multiple test correction (adjusted $P < 0.01$) were performed to identify significantly differently edited sites across the samples (absolute editing difference > 10%). Differential expressed genes (DEGs) were identified using DESeq2[16] with default settings (FDR cut-off = 1e-3). GOrilla[17] was used to identify enriched GO terms in up-/downregulated genes (FDR q-value < 1e-5, enrichment > 1.5).

Fluorescence microscopy. Oligonucleotides used in fluorescence microscopy experiments were purchased with a C6 5'-aminolinker. Oligonucleotides were modified with an Atto488 fluorescent dye and precipitated subsequently to remove the remaining dye as described recently[5]. 24 h post transfection, samples were prepared for fluorescence microscopy and images captured and processed as described previously in detail[5].

Western Blot. Western Blot was performed as described recently in Hanswillemenke et al. 2024[5]. Briefly, cells were harvested 24 h post transfection in RIPA buffer, incubated 15 min on ice and centrifuged for 30 min at 4°C. Equal protein amounts from the supernatant were loaded on a Novex 8-16% Tris-Glycine Mini Gel (Thermo Fisher Scientific) and subsequently transferred on a PVDF membrane using wet transfer. Membranes were washed once with TBST and blocked with 5% non-fat dry milk in TBST for 1 h at room temperature. Primary antibodies (α-NONO: sc-376865, Santa Cruz Biotechnology; α-GAPDH: MA5-15738, Thermo Fisher Scientific) were diluted 1:1,000 in 2% non-fat dry milk in TBST and incubated overnight at 4°C. Afterwards, the membrane was washed three times and incubated for 1 h at room temperature with secondary antibody (115-035-003, Jackson Immuno Research Laboratories) diluted 1:10,000 in 2% non-fat dry milk in TBST. The membrane was washed three times with TBST and imaged using an Odyssey Fc Imaging system (LiCOR) by adding 1x ECL substrate plus 0.03% H_2O_2.

isASO-ID. A detailed protocol for the isASO-ID experiment can be found in Hanswillemenke et al. 2024[5]. Briefly, Hela cells were SILAC-labeled with DMEM supplemented with dialyzed FBS and $^{12}C_6$, $^{14}N_2$ L-lysine (Sigma Aldrich) and $^{12}C_6$, $^{14}N_4$ L-arginine (Sigma Aldrich) in the 'light' condition, D_4 L-lysine (Cabridge Isotope Laboratories) and $^{13}C_6$ L-arginine (Silantes) in the 'medium' condition and $^{13}C_6$, $^{15}N_2$ L-lysine

(Cambridge Isotope Laboratories) and $^{13}C_6$ $^{15}N_4$ L-arginine (Silantes) were added to the 'heavy' medium. Cells were cultured for at least 10 days in SILAC medium before the experiment.

For each condition, four wells of a 6-well plate were seeded (400,000 cells/well) per condition and transfected 24 h later with 10 µl Lipofectamine RNAimax per well. 24 h post transfection, cells were washed with PBS, fixed with, washed with PBS and permeabilized gently with Triton X-100. Cells were washed twice with PBS before 240 nM purified SNAP-BASU-His in conjugation buffer (2.5% glycerol, 50 mM NaCl, 10 mM Tris-HCl pH 8.0, 1 mM dithiothreitol) was incubated on the cells for 1 h at 37 °C. Unconjugated enzyme was washed away twice with PBS and biotinylation induced by addition of biotinylation buffer. After 24 h, cells were washed twice with PBS, transferred to ice and harvested in 225 µl 1x Laemmli buffer per well using a cell scraper. To reverse fixation, cells were boiled subsequently for 1 h at 98°C. Before streptavidin pulldown, the lysate was diluted 4-fold in PBS.

For streptavidin pulldown, equal amounts from all three conditions (light, medium, heavy) were pooled and incubated overnight at 4 °C with constant rotation on 200 µl Streptavidin Magnetic Sepharose (GE Healthcare). The next day, the 'harsh wash' protocol[5] was applied. Proteins were eluted twice with 20 µl elution buffer (10 mM Tris, pH 7.4, 2% SDS, 5% β-mercaptoethanol and 2 mM biotin) and heating to 95°C for 15 min. Samples were loaded with Laemmli buffer on a Novex 8–16% Tris-Glycine Mini Gel (Thermo Fisher Scientific) and run approx. 2-3 cm into the gel. Gels were stained with ReadyBlue Protein Gel Stain (Sigma Aldrich) for 2 h and destained with water overnight.

In-gel digest and MS: Sample processing for mass spectrometry was performed as described recently[5]. After destaining, the whole lanes of each sample was excised and the intense Streptavidin band (approx. 17 kDa) separated and measured independently. The proteins were digested in-gel with trypsin[18] and desalted using StageTips[19]. Peptide mixtures were measured on an Easy-nLC 1200 coupled to a Q Exactive HF mass spectrometer (both Thermo Fisher Scientific) as described previously[20] with minor modifications: peptide mixtures were separated with a 57-min segmented gradient of 10%-33%-50%-90% of HPLC solvent B (80 acetonitrile in 0.1% formic acid) in HPLC solvent A (0.1% formic acid) at a flow rate of 200 nl min^{-1}. The seven most intense precursor ions were selected in each cycle and sequentially fragmented using higher-energy collisional dissociation (HCD). Sequenced precursor masses were excluded from further selection for 30 s. For MS/MS fragmentation and the MS scan, target values were 10^5 and 3×10^6 charges, respectively.

After the measurement, the acquired MS spectra were analyzed with the MaxQuant software package version 2.5.0.0 with the integrated Andromeda search engine[21, 22]. A database search against a target-decoy *Homo sapiens* database obtained from UniProt (downloaded 30 January 2024) was performed and commonly observed contaminants were rejected. In addition, data were searched against SNAP-BASU-His. Full trypsin digestion was specificity was required for database search and up to two missed cleavages were allowed. N-terminal protein acetylation and methionine oxidation were set as variable modifications and carbamidomethylation of cysteine was set as fixed modification. Initial precursor mass tolerance was set to 4.5 ppm and 20 ppm at the MS/MS level. Peptides labeled containing Lys4/Arg6 and Lys8/Arg10 were defined as medium and heavy, respectively. Only protein groups with two or more quantified peptides were considered for quantification. The false discovery rate (FDR) for peptides, proteins and modifications was set to 0.01, estimated by the target-decoy approach[23]. Scatter plots were created with Perseus software[24] (version 1.5.15.0) and modified with CorelDraw 2017.

Supplementary Tables

Supplementary Table 1 | Primer List. Sequences are depicted in 5' → 3' orientation.

Target site	Forward primer	Reverse primer	Sequencing primer
GAPDH ORF	CTCAAGATCATCAGCAATGCCTCCTGC	GAGCACAGGGTACTTTATTGATGGTACATGACAAGG	GCTGTTGAAGTCAGAGGAGACC
GAPDH ORF mRNA	CTCAAGATCATCAGCAATGCCTCCTGC	AGCGTCAAAGGTGGAGGAGTGG	GCTGTTGAAGTCAGAGGAGACC
Long/young & Short/old GAPDH ORF pre-mRNA	CCCGCTCCCTCTTTCTTTGCAG	AGCGTCAAAGGTGGAGGAGTGG	GCTGTTGAAGTCAGAGGAGACC
MECP2 W104X (murine)	GGAGGAAAAGTCAGAAGACC	AAGGAGGTGTCTCCCACCTT	Rev. primer
SERPINA E342K (PiZZ), in vitro	ACCCACGATATCATCACCAA	CTTTTCCCATGAAGAGGGGAG	ACTTGGTATTTTGTTCAATC
SERPINA E342K (PiZZ), in vivo	CTGGAACCTATGATCTGAAGAGCGT	GTGGGATTCACCACTTTTCCCATG	Rev. primer
STAT1 Y701C	GCTTCATCAGCAAGGAGCGAGAGCG	CTTCAGACACAGAAATCAACTCAGTC	GGCTGCTGAGAATATTCCTGAGAATC
Long/young STAT1 Y701C pre-mRNA	TTCTGAAACTCCACCCATGC	CTTCAGACACAGAAATCAACTCAGTC	GAAAGCTCCTAGCTAGTGTCC
Short/old STAT1 Y701C pre-mRNA	GGCTGCTGAGAATATTCCTGAGAATC	ACAGTGTATCTCAATCATTACTTTGGCC	Fwd. primer
ACTB 3'UTR (from ref.[25])	CGAGCATCCCCCAAAGTTCAC	CACTCCCAGGGAGACCAAAAGC	Rev. primer
Short/old ACTB 3'UTR (from ref.[25]) pre-mRNA	GGAGCTGTCACATCCAGGGTC	CACTCCCAGGGAGACCAAAAGC	Rev. primer
LRRK2 G2019S	CTGGGTTGCTGGAGATTGAT	GGTGTGCCCTCTGATGTTTT	ACCCTACAGCACAGGATTGC

Supplementary Figures (continued)

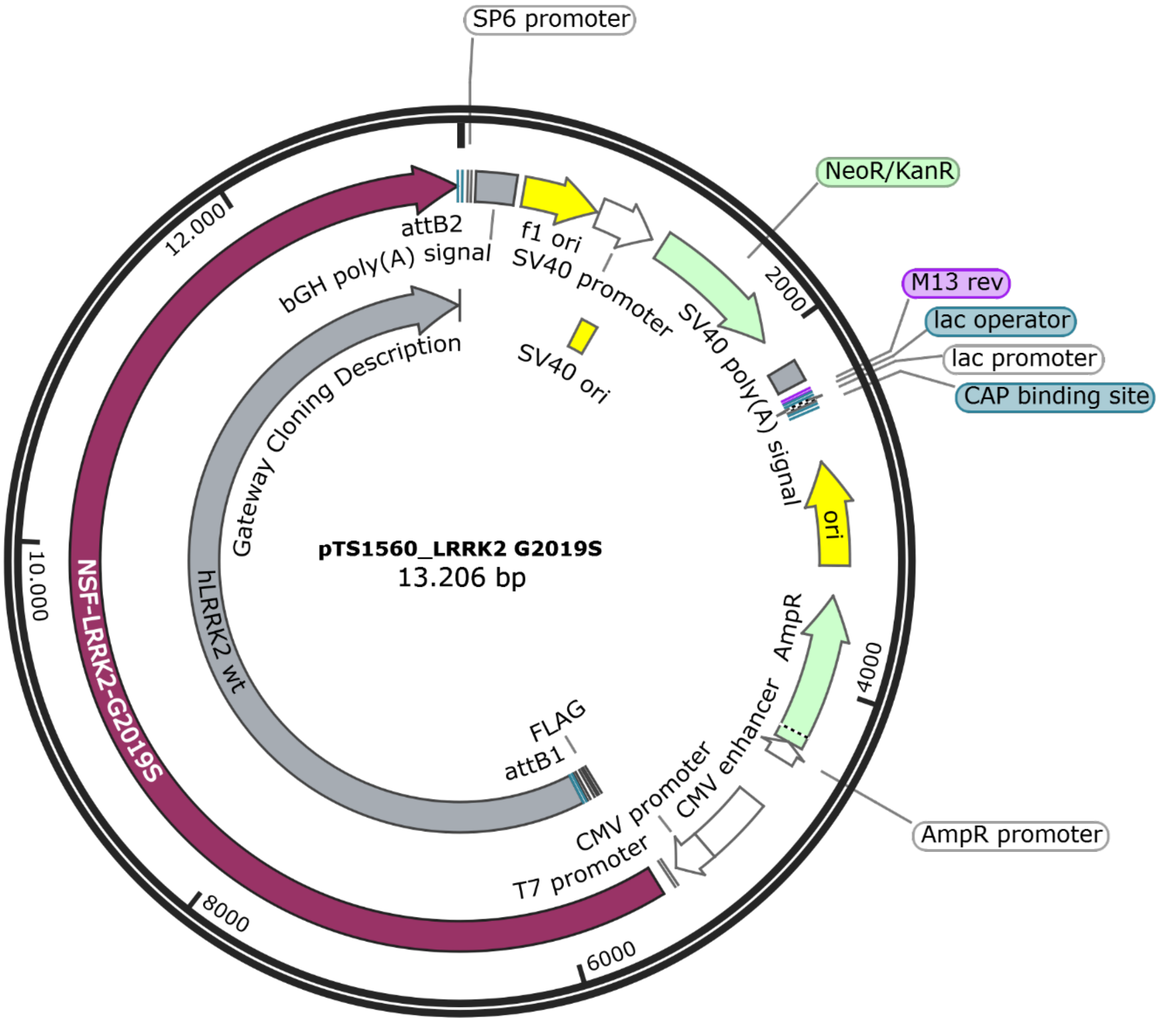

Supplementary Figure 9 | A map of the LRRK2 G2019S-encoding plasmid (pTS1560). This plasmid was used to generate the data shown in Supplementary Figure 4F. The human LRRK2 G2019S gene is continuously expressed under the control of a CMV promoter.

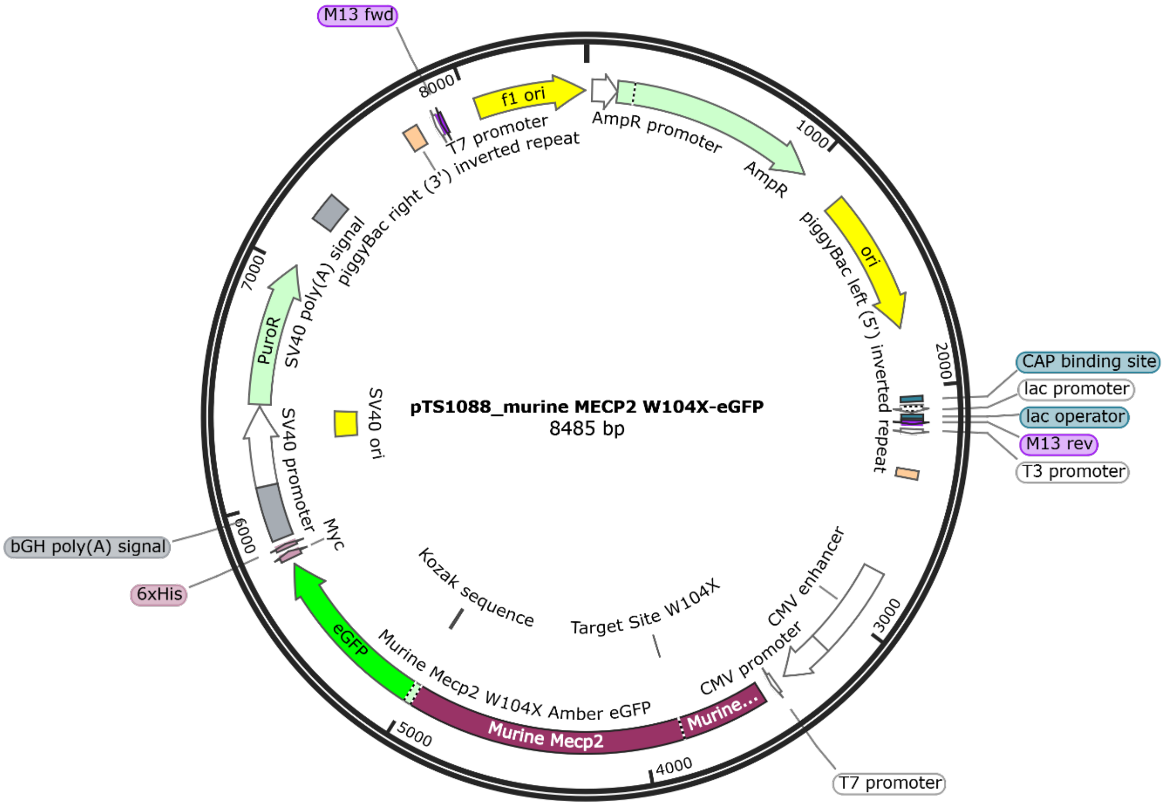

Supplementary Figure 10 | Map of the murine MECP2 W104X-encoding plasmid (pTS1088). This plasmid was used to genomically integrate the murine MECP2 W104X gene into HeLa cells via the piggyBac transposase system for the data shown in Supplementary Figure 3A. The murine MECP2 W104X gene is also C-terminally tagged with eGFP and is continuously expressed under the control of a CMV promoter.

Supplementary Sequence 1 | Reference sequence and editing site of the human GAPDH ORF target transcript (coding region of NCBI accession number NM_002046). Target adenosine is highlighted in red.

```
1        ATGGGGAAGG TGAAGGTCGG AGTCAACGGA TTTGGTCGTA TTGGGCGCCT GGTCACCAGG
61       GCTGCTTTTA ACTCTGGTAA AGTGGATATT GTTGCCATCA ATGACCCCTT CATTGACCTC
121      AACTACATGG TTTACATGTT CCAATATGAT TCCACCCATG GCAAATTCCA TGGCACCGTC
181      AAGGCTGAGA ACGGGAAGCT TGTCATCAAT GGAAATCCCA TCACCATCTT CCAGGAGCGA
241      GATCCCTCCA AAATCAAGTG GGGCGATGCT GGCGCTGAGT ACGTCGTGGA GTCCACTGGC
301      GTCTTCACCA CCATGGAGAA GGCTGGGGCT CATTTGCAGG GGGGAGCCAA AAGGGTCATC
361      ATCTCTGCCC CCTCTGCTGA TGCCCCCATG TTCGTCATGG GTGTGAACCA TGAGAAGTAT
421      GACAACAGCC TCAAGATCAT CAGCAATGCC TCCTGCACCA CCAACTGCTT AGCACCCCTG
481      GCCAAGGTCA TCCATGACAA CTTTGGTATC GTGGAAGGAC TCATGACCAC AGTCCATGCC
541      ATCACTGCCA CCCAGAAGAC TGTGGATGGC CCCTCCGGGA AACTGTGGCG TGATGGCCGC
601      GGGGCTCTCC AGAACATCAT CCCTGCCTCT ACTGGCGCTG CCAAGGCTGT GGGCAAGGTC
661      ATCCCTGAGC TGAACGGGAA GCTCACTGGC ATGGCCTTCC GTGTCCCCAC TGCCAACGTG
721      TCAGTGGTGG ACCTGACCTG CCGTCTAGAA AAACCTGCCA AATATGATGA CATCAAGAAG
781      GTGGTGAAGC AGGCGTCGGA GGGCCCCCTC AAGGGCATCC TGGGCTACAC TGAGCACCAG
841      GTGGTCTCCT CTGACTTCAA CAGCGACACC CACTCCTCCA CCTTTGACGC TGGGGCTGGC
901      ATTGCCCTCA ACGACCACTT TGTCAAGCTC ATTTCCTGGT ATGACAACGA ATTTGGCTAC
961      AGCAACAGGG TGGTGGACCT CATGGCCCAC ATGGCCTCCA AGGAGTAA
```

Supplementary Sequence 2 | Reference sequence and editing site of the human SERPINA1 E342K target transcript genomically integrated into HeLa cells as described in ref.[7]. Target adenosine is highlighted in red.

```
1        ATGCCGTCTT CTGTCTCGTG GGGCATCCTC CTGCTGGCAG GCCTGTGCTG CCTGGTCCCT
61       GTCTCCCTGG CTGAGGATCC CCAGGGAGAT GCTGCCCAGA AGACAGATAC ATCCCACCAT
121      GATCAGGATC ACCCAACCTT CAACAAGATC ACCCCCAACC TGGCTGAGTT CGCCTTCAGC
181      CTATACCGCC AGCTGGCACA CCAGTCCAAC AGCACCAATA TCTTCTTCTC CCCAGTGAGC
241      ATCGCTACAG CCTTTGCAAT GCTCTCCCTG GGGACCAAGG CTGACACTCA CGATGAAATC
301      CTGGAGGGCC TGAATTTCAA CCTCACGGAG ATTCCGGAGG CTCAGATCCA TGAAGGCTTC
361      CAGGAACTCC TCCGTACCCT CAACCAGCCA GACAGCCAGC TCCAGCTGAC CACCGGCAAT
421      GGCCTGTTCC TCAGCGAGGG CCTGAAGCTA GTGGATAAGT TTTTGGAGGA TGTTAAAAAG
481      TTGTACCACT CAGAAGCCTT CACTGTCAAC TTCGGGGACA CCGAAGAGGC CAAGAAACAG
541      ATCAACGATT ACGTGGAGAA GGGTACTCAA GGGAAAATTG TGGATTTGGT CAAGGAGCTT
601      GACAGAGACA CAGTTTTTGC TCTGGTGAAT TACATCTTCT TTAAAGGCAA ATGGGAGAGA
661      CCCTTTGAAG TCAAGGACAC CGAGGAAGAG GACTTCCACG TGGACCAGGT GACCACCGTG
721      AAGGTGCCTA TGATGAAGCG TTTAGGCATG TTTAACATCC AGCACTGTAA GAAGCTGTCC
781      AGCTGGGTGC TGCTGATGAA ATACCTGGGC AATGCCACCG CCATCTTCTT CCTGCCTGAT
841      GAGGGGAAAC TACAGCACCT GGAAAATGAA CTCACCCACG ATATCATCAC CAAGTTCCTG
901      GAAAATGAAG ACAGAAGGTC TGCCAGCTTA CATTTACCCA AACTGTCCAT TACTGGAACC
961      TATGATCTGA AGAGCGTCCT GGGTCAACTG GGCATCACTA AGGTCTTCAG CAATGGGGCT
1021     GACCTCTCCG GGGTCACAGA GGAGGCACCC CTGAAGCTCT CCAAGGCCGT GCATAAGGCT
1081     GTGCTGACCA TCGACAAGAA AGGGACTGAA GCTGCTGGGG CCATGTTTTT AGAGGCCATA
1141     CCCATGTCTA TCCCCCCCGA GGTCAAGTTC AACAAACCCT TTGTCTTCTT AATGATTGAA
1201     CAAAATACCA AGTCTCCCCT CTTCATGGGA AAAGTGGTGA ATCCCACCCA AAAATAA
```

Supplementary Sequence 3 | Reference sequence and editing site of the human STAT1 Y701 target transcript (coding region of NCBI accession number NM_007315). Target adenosine is highlighted in red.

1	ATGTCTCAGT GGTACGAACT TCAGCAGCTT GACTCAAAAT TCCTGGAGCA GGTTCACCAG
61	CTTTATGATG ACAGTTTTCC CATGGAAATC AGACAGTACC TGGCACAGTG GTTAGAAAAG
121	CAAGACTGGG AGCACGCTGC CAATGATGTT TCATTTGCCA CCATCCGTTT TCATGACCTC
181	CTGTCACAGC TGGATGATCA ATATAGTCGC TTTTCTTTGG AGAATAACTT CTTGCTACAG
241	CATAACATAA GGAAAAGCAA GCGTAATCTT CAGGATAATT TTCAGGAAGA CCCAATCCAG
301	ATGTCTATGA TCATTTACAG CTGTCTGAAG GAAGAAAGGA AAATTCTGGA AAACGCCCAG
361	AGATTTAATC AGGCTCAGTC GGGGAATATT CAGAGCACAG TGATGTTAGA CAAACAGAAA
421	GAGCTTGACA GTAAAGTCAG AAATGTGAAG GACAAGGTTA TGTGTATAGA GCATGAAATC
481	AAGAGCCTGG AAGATTTACA AGATGAATAT GACTTCAAAT GCAAAACCTT GCAGAACAGA
541	GAACACGAGA CCAATGGTGT GGCAAAGAGT GATCAGAAAC AAGAACAGCT GTTACTCAAG
601	AAGATGTATT TAATGCTTGA CAATAAGAGA AAGGAAGTAG TTCACAAAAT AATAGAGTTG
661	CTGAATGTCA CTGAACTTAC CCAGAATGCC CTGATTAATG ATGAACTAGT GGAGTGGAAG
721	CGGAGACAGC AGAGCGCCTG TATTGGGGGG CCGCCCAATG CTTGCTTGGA TCAGCTGCAG
781	AACTGGTTCA CTATAGTTGC GGAGAGTCTG CAGCAAGTTC GGCAGCAGCT TAAAAAGTTG
841	GAGGAATTGG AACAGAAATA CACCTACGAA CATGACCCTA TCACAAAAAA CAAACAAGTG
901	TTATGGGACC GCACCTTCAG TCTTTTCCAG CAGCTCATTC AGAGCTCGTT TGTGGTGGAA
961	AGACAGCCCT GCATGCCAAC GCACCCTCAG AGGCCGCTGG TCTTGAAGAC AGGGGTCCAG
1021	TTCACTGTGA AGTTGAGACT GTTGGTGAAA TTGCAAGAGC TGAATTATAA TTTGAAAGTC
1081	AAAGTCTTAT TTGATAAAGA TGTGAATGAG AGAAATACAG TAAAAGGATT TAGGAAGTTC
1141	AACATTTTGG GCACGCACAC AAAAGTGATG AACATGGAGG AGTCCACCAA TGGCAGTCTG
1201	GCGGCTGAAT TTCGGCACCT GCAATTGAAA GAACAGAAAA ATGCTGGCAC CAGAACGAAT
1261	GAGGGTCCTC TCATCGTTAC TGAAGAGCTT CACTCCCTTA GTTTTGAAAC CCAATTGTGC
1321	CAGCCTGGTT TGGTAATTGA CCTCGAGACG ACCTCTCTGC CCGTTGTGGT GATCTCCAAC
1381	GTCAGCCAGC TCCCGAGCGG TTGGGCCTCC ATCCTTTGGT ACAACATGCT GGTGGCGGAA
1441	CCCAGGAATC TGTCCTTCTT CCTGACTCCA CCATGTGCAC GATGGGCTCA GCTTTCAGAA
1501	GTGCTGAGTT GGCAGTTTTC TTCTGTCACC AAAAGAGGTC TCAATGTGGA CCAGCTGAAC
1561	ATGTTGGGAG AGAAGCTTCT TGGTCCTAAC GCCAGCCCCG ATGGTCTCAT TCCGTGGACG
1621	AGGTTTTGTA AGGAAAATAT AAATGATAAA AATTTTCCCT TCTGGCTTTG GATTGAAAGC
1681	ATCCTAGAAC TCATTAAAAA ACACCTGCTC CCTCTCTGGA ATGATGGGTG CATCATGGGC
1741	TTCATCAGCA AGGAGCGAGA GCGTGCCCTG TTGAAGGACC AGCAGCCGGG GACCTTCCTG
1801	CTGCGGTTCA GTGAGAGCTC CCGGGAAGGG GCCATCACAT TCACATGGGT GGAGCGGTCC
1861	CAGAACGGAG GCGAACCTGA CTTCCATGCG GTTGAACCCT ACACGAAGAA AGAACTTTCT
1921	GCTGTTACTT TCCCTGACAT CATTCGCAAT TACAAAGTCA TGGCTGCTGA GAATATTCCT
1981	GAGAATCCCC TGAAGTATCT GTATCCAAAT ATTGACAAAG ACCATGCCTT TGGAAAGTAT
2041	TACTCCAGGC CAAAGGAAGC ACCAGAGCCA ATGGAACTTG ATGGCCCTAA AGGAACTGGA
2101	T**A**TATCAAGA CTGAGTTGAT TTCTGTGTCT GAAGTTCACC CTTCTAGACT TCAGACCACA
2161	GACAACCTGC TCCCCATGTC TCCTGAGGAG TTTGACGAGG TGTCTCGGAT AGTGGGCTCT
2221	GTAGAATTCG ACAGTATGAT GAACACAGTA TAG

Supplementary Sequence 4 | Reference sequence and editing site of the human ACTB 3'-UTR target transcript (NCBI accession number NM_001101). Target adenosine is highlighted in red.

1	ACCGCCGAGA CCGCGTCCGC CCCGCGAGCA CAGAGCCTCG CCTTTGCCGA TCCGCCGCCC
61	GTCCACACCC GCCGCCAGCT CACCATGGAT GATGATATCG CCGCGCTCGT CGTCGACAAC
121	GGCTCCGGCA TGTGCAAGGC CGGCTTCGCG GGCGACGATG CCCCCCGGGC CGTCTTCCCC
181	TCCATCGTGG GGCGCCCCAG GCACCAGGGC GTGATGGTGG GCATGGGTCA GAAGGATTCC
241	TATGTGGGCG ACGAGGCCCA GAGCAAGAGA GGCATCCTCA CCCTGAAGTA CCCCATCGAG
301	CACGGCATCG TCACCAACTG GGACGACATG GAGAAAATCT GGCACCACAC CTTCTACAAT
361	GAGCTGCGTG TGGCTCCCGA GGAGCACCCC GTGCTGCTGA CCGAGGCCCC CCTGAACCCC
421	AAGGCCAACC GCGAGAAGAT GACCCAGATC ATGTTTGAGA CCTTCAACAC CCCAGCCATG
481	TACGTTGCTA TCCAGGCTGT GCTATCCCTG TACGCCTCTG GCCGTACCAC TGGCATCGTG
541	ATGGACTCCG GTGACGGGGT CACCCACACT GTGCCCATCT ACGAGGGGTA TGCCCTCCCC

```
601       CATGCCATCC TGCGTCTGGA CCTGGCTGGC CGGGACCTGA CTGACTACCT CATGAAGATC
661       CTCACCGAGC GCGGCTACAG CTTCACCACC ACGGCCGAGC GGGAAATCGT GCGTGACATT
721       AAGGAGAAGC TGTGCTACGT CGCCCTGGAC TTCGAGCAAG AGATGGCCAC GGCTGCTTCC
781       AGCTCCTCCC TGGAGAAGAG CTACGAGCTG CCTGACGGCC AGGTCATCAC CATTGGCAAT
841       GAGCGGTTCC GCTGCCCTGA GGCACTCTTC CAGCCTTCCT TCCTGGGCAT GGAGTCCTGT
901       GGCATCCACG AAACTACCTT CAACTCCATC ATGAAGTGTG ACGTGGACAT CCGCAAAGAC
961       CTGTACGCCA ACACAGTGCT GTCTGGCGGC ACCACCATGT ACCCTGGCAT TGCCGACAGG
1021      ATGCAGAAGG AGATCACTGC CCTGGCACCC AGCACAATGA AGATCAAGAT CATTGCTCCT
1081      CCTGAGCGCA AGTACTCCGT GTGGATCGGC GGCTCCATCC TGGCCTCGCT GTCCACCTTC
1141      CAGCAGATGT GGATCAGCAA GCAGGAGTAT GACGAGTCCG GCCCCTCCAT CGTCCACCGC
1201      AAATGCTTCT AGGCGGACTA TGACTTAGTT GCGTTACACC CTTTCTTGAC AAAACCTAAC
1261      TTGCGCAGAA AACAAGATGA GATTGGCATG GCTTTATTTG TTTTTTTTGT TTTGTTTTGG
1321      TTTTTTTTTT TTTTTTGGCT TGACTCAGGA TTTAAAAACT GGAACGGTGA AGGTGACAGC
1381      AGTCGGTTGG AGCGAGCATC CCCCAAAGTT CACAATGTGG CCGAGGACTT TGATTGCACA
1441      TTGTTGTTTT TTTAATAGTC ATTCCAAATA TGAGATGCGT TGTTACAGGA AGTCCCTTGC
1501      CATCCTAAAA GCCACCCCAC TTCTCTCTAA GGAGAATGGC CCAGTCCTCT CCCAAGTCCA
1561      CACAGGGGAG GTGATAGCAT TGCTTTCGTG TAAATTATGT AATGCAAAAT TTTTTTAATC
1621      TTCGCCTTAA TACTTTTTTA TTTTGTTTTA TTTTGAATGA TGAGCCTTCG TGCCCCCCCT
1681      TCCCCCTTTT TTGTCCCCCA ACTTGAGATG TATGAAGGCT TTTGGTCTCC CTGGGAGTGG
1741      GTGGAGGCAG CCAGGGCTTA CCTGTACACT GACTTGAGAC CAGTTGAATA AAAGTGCACA
1801      CCTTAAAAAT GA
```

Supplementary Sequence 5 | Reference sequence and editing site of the human LRRK2 G2019S cDNA construct on plasmid pTS1560 (Supplementary Note 1). Target adenosine is highlighted in red.

```
1         ATGGATTATA AAGATGATGA TGATAAAGGG TCGGCCGCCA GCTGGAGCCA CCCTCAGTTC
61        GAGAAGGGAG GAGGAAGCGG CGGAGGCAGC GGAGGAGGAA GCTGGAGCCA CCCGCAGTTC
121       GAGAAAGGAG CTAGATCAAC AAGTTTGTAC AAAAAAGCAG GCACCATGGC TAGTGGCAGC
181       TGTCAGGGGT GCGAAGAGGA CGAGGAAACT CTGAAGAAGT TGATAGTCAG GCTGAACAAT
241       GTCCAGGAAG GAAAACAGAT AGAAACGCTG GTCCAAATCC TGGAGGATCT GCTGGTGTTC
301       ACGTACTCCG AGCACGCCTC CAAGTTATTT CAAGGCAAAA ATATCCATGT GCCTCTGTTG
361       ATCGTCTTGG ACTCCTATAT GAGAGTCGCG AGTGTGCAGC AGGTGGGTTG GTCACTTCTG
421       TGCAAATTAA TAGAAGTCTG TCCAGGTACA ATGCAAAGCT TAATGGGACC CCAGGATGTT
481       GGAAATGATT GGGAAGTCCT TGGTGTTCAC CAATTGATTC TTAAAATGCT AACAGTTCAT
541       AATGCCAGTG TAAACTTGTC AGTGATTGGA CTGAAGACCT TAGATCTCCT CCTAACTTCA
601       GGTAAAATCA CCTTGCTGAT ATTGGATGAA GAAAGTGATA TTTTCATGTT AATTTTTGAT
661       GCCATGCACT CATTTCCAGC CAATGATGAA GTCCAGAAAC TTGGATGCAA AGCTTTACAT
721       GTGCTGTTTG AGAGAGTCTC AGAGGAGCAA CTGACTGAAT TTGTTGAGAA CAAAGATTAT
781       ATGATATTGT TAAGTGCGCT AACAAATTTT AAAGATGAAG AGGAAATTGT GCTTCATGTG
841       CTGCATTGTT TACATTCCCT AGCGATTCCT TGCAATAATG TGGAAGTCCT CATGAGTGGC
901       AATGTCAGGT GTTATAATAT TGTGGTGGAA GCTATGAAAG CATTCCCTAT GAGTGAAAGA
961       ATTCAAGAAG TGAGTTGCTG TTTGCTCCAT AGGCTTACAT TAGGTAATTT TTTCAATATC
1021      CTGGTATTAA ACGAAGTCCA TGAGTTTGTG GTGAAAGCTG TGCAGCAGTA CCCAGAGAAT
1081      GCAGCATTGC AGATCTCAGC GCTCAGCTGT TTGGCCCTCC TCACTGAGAC TATTTTCTTA
1141      AATCAAGATT TAGAGGAAAA GAATGAGAAT CAAGAGAATG ATGATGAGGG GGAAGAAGAT
1201      AAATTGTTTT GGCTGGAAGC CTGTTACAAA GCATTAACGT GGCATAGAAA GAACAAGCAC
1261      GTGCAGGAGG CCGCATGCTG GGCACTAAAT AATCTCCTTA TGTACCAAAA CAGTTTACAT
1321      GAGAAGATTG GAGATGAAGA TGGCCATTTC CCAGCTCATA GGGAAGTGAT GCTCTCCATG
1381      CTGATGCATT CTTCATCAAA GGAAGTTTTC CAGGCATCTG CGAATGCATT GTCAACTCTC
1441      TTAGAACAAA ATGTTAATTT CAGAAAAATA CTGTTATCAA AAGGAATACA CCTGAATGTT
1501      TTGGAGTTAA TGCAGAAGCA TATACATTCT CCTGAAGTGG CTGAAAGTGG CTGTAAAATG
1561      CTAAATCATC TTTTTGAAGG AAGCAACACT TCCCTGGATA TAATGGCAGC AGTGGTCCCC
1621      AAAATACTAA CAGTTATGAA ACGTCATGAG ACATCATTAC CAGTGCAGCT GGAGGCGCTT
1681      CGAGCTATTT TACATTTTAT AGTGCCTGGC ATGCCAGAAG AATCCAGGGA GGATACAGAA
1741      TTTCATCATA AGCTAAATAT GGTTAAAAAA CAGTGTTTCA AGAATGATAT TCACAAACTG
```

1801 GTCCTAGCAG CTTTGAACAG GTTCATTGGA AATCCTGGGA TTCAGAAATG TGGATTAAAA
1861 GTAATTTCTT CTATTGTACA TTTTCCTGAT GCATTAGAGA TGTTATCCCT GGAAGGTGCT
1921 ATGGATTCAG TGCTTCACAC ACTGCAGATG TATCCAGATG ACCAAGAAAT TCAGTGTCTG
1981 GGTTTAAGTC TTATAGGATA CTTGATTACA AAGAAGAATG TGTTCATAGG AACTGGACAT
2041 CTGCTGGCAA AAATTCTGGT TTCCAGCTTA TACCGATTTA AGGATGTTGC TGAAATACAG
2101 ACTAAAGGAT TTCAGACAAT CTTAGCAATC CTCAAATTGT CAGCATCTTT TTCTAAGCTG
2161 CTGGTGCATC ATTCATTTGA CTTAGTAATA TTCCATCAAA TGTCTTCCAA TATCATGGAA
2221 CAAAAGGATC AACAGTTTCT AAACCTCTGT TGCAAGTGTT TTGCAAAAGT AGCTATGGAT
2281 GATTACTTAA AAAATGTGAT GCTAGAGAGA GCGTGTGATC AGAATAACAG CATCATGGTT
2341 GAATGCTTGC TTCTATTGGG AGCAGATGCC AATCAAGCAA AGGAGGGATC TTCTTTAATT
2401 TGTCAGGTAT GTGAGAAAGA GAGCAGTCCC AAATTGGTGG AACTCTTACT GAATAGTGGA
2461 TCTCGTGAAC AAGATGTACG AAAAGCGTTG ACGATAAGCA TTGGGAAAGG TGACAGCCAG
2521 ATCATCAGCT TGCTCTTAAG GAGGCTGGCC CTGGATGTGG CCAACAATAG CATTTGCCTT
2581 GGAGGATTTT GTATAGGAAA AGTTGAACCT TCTTGGCTTG GTCCTTTATT TCCAGATAAG
2641 ACTTCTAATT TAAGGAAACA AACAAATATA GCATCTACAC TAGCAAGAAT GGTGATCAGA
2701 TATCAGATGA AAAGTGCTGT GGAAGAAGGA ACAGCCTCAG GCAGCGATGG AAATTTTTCT
2761 GAAGATGTGC TGTCTAAATT TGATGAATGG ACCTTTATTC CTGACTCTTC TATGGACAGT
2821 GTGTTTGCTC AAAGTGATGA CCTGGATAGT GAAGGAAGTG AAGGCTCATT TCTTGTGAAA
2881 AAGAAATCTA ATTCAATTAG TGTAGGAGAA TTTTACCGAG ATGCCGTATT ACAGCGTTGC
2941 TCACCAAATT TGCAAAGACA TTCCAATTCC TTGGGGCCCA TTTTTGATCA TGAAGATTTA
3001 CTGAAGCGAA AAAGAAAAAT ATTATCTTCA GATGATTCAC TCAGGTCATC AAAACTTCAA
3061 TCCCATATGA GGCATTCAGA CAGCATTTCT TCTCTGGCTT CTGAGAGAGA ATATATTACA
3121 TCACTAGACC TTTCAGCAAA TGAACTAAGA GATATTGATG CCCTAAGCCA GAAATGCTGT
3181 ATAAGTGTTC ATTTGGAGCA TCTTGAAAAG CTGGAGCTTC ACCAGAATGC ACTCACGAGC
3241 TTTCCACAAC AGCTATGTGA AACTCTGAAG AGTTTGACAC ATTTGGACTT GCACAGTAAT
3301 AAATTTACAT CATTTCCTTC TTATTTGTTG AAAATGAGTT GTATTGCTAA TCTTGATGTC
3361 TCTCGAAATG ACATTGGACC CTCAGTGGTT TTAGATCCTA CAGTGAAATG TCCAACTCTG
3421 AAACAGTTTA ACCTGTCATA TAACCAGCTG TCTTTTGTAC CTGAGAACCT CACTGATGTG
3481 GTAGAGAAAC TGGAGCAGCT CATTTTAGAA GGAAATAAAA TATCAGGGAT ATGCTCCCCC
3541 TTGAGACTGA AGGAACTGAA GATTTTAAAC CTTAGTAAGA ACCACATTTC ATCCCTATCA
3601 GAGAACTTTC TTGAGGCTTG TCCTAAAGTG GAGAGTTTCA GTGCCAGAAT GAATTTTCTT
3661 GCTGCTATGC CTTTCTTGCC TCCTTCTATG ACAATCCTAA AATTATCTCA GAACAAATTT
3721 TCCTGTATTC CAGAAGCAAT TTTAAATCTT CCACACTTGC GGTCTTTAGA TATGAGCAGC
3781 AATGATATTC AGTACCTACC AGGTCCCGCA CACTGGAAAT CTTTGAACTT AAGGGAACTC
3841 TTATTTAGCC ATAATCAGAT CAGCATCTTG GACTTGAGTG AAAAAGCATA TTTATGGTCT
3901 AGAGTAGAGA AACTGCATCT TTCTCACAAT AAACTGAAAG AGATTCCTCC TGAGATTGGC
3961 TGTCTTGAAA ATCTGACATC TCTGGATGTC AGTTACAACT TGGAACTAAG ATCCTTTCCC
4021 AATGAAATGG GGAAATTAAG CAAAATATGG GATCTTCCTT TGGATGAACT GCATCTTAAC
4081 TTTGATTTTA AACATATAGG ATGTAAAGCC AAAGACATCA TAAGGTTTCT TCAACAGCGA
4141 TTAAAAAAGG CTGTGCCTTA TAACCGAATG AAACTTATGA TTGTGGGAAA TACTGGGAGT
4201 GGTAAAACCA CCTTATTGCA GCAATTAATG AAAACCAAGA AATCAGATCT TGGAATGCAA
4261 AGTGCCACAG TTGGCATAGA TGTGAAAGAC TGGCCTATCC AAATAAGAGA CAAAAGAAAG
4321 AGAGATCTCG TCCTAAATGT GTGGGATTTT GCAGGTCGTG AGGAATTCTA TAGTACTCAT
4381 CCCCATTTTA TGACGCAGCG AGCATTGTAC CTTGCTGTCT ATGACCTCAG CAAAGGACAG
4441 GCTGAAGTTG ATGCCATGAA GCCTTGGCTC TTCAATATAA AGGCTCGCGC TTCTTCTTCC
4501 CCTGTGATTC TCGTTGGCAC ACATTTGGAT GTTTCTGATG AGAAGCAACG CAAAGCCTGC
4561 ATGAGTAAAA TCACCAAGGA ACTCCTGAAT AAGCGAGGGT TCCCTGCCAT ACGAGATTAC
4621 CACTTTGTGA ATGCCACCGA GGAATCTGAT GCTTTGGCAA AACTTCGGAA AACCATCATA
4681 AACGAGAGCC TTAATTTCAA GATCCGAGAT CAGCTTGTTG TTGGACAGCT GATTCCAGAC
4741 TGCTATGTAG AACTTGAAAA AATCATTTTA TCGGAGCGTA AAAATGTGCC AATTGAATTT
4801 CCCGTAATTG ACCGGAAACG ATTATTACAA CTAGTGAGAG AAAATCAGCT GCAGTTAGAT
4861 GAAAATGAGC TTCCTCACGC AGTTCACTTT CTAAATGAAT CAGGAGTCCT TCTTCATTTT
4921 CAAGACCCAG CACTGCAGTT AAGTGACTTG TACTTTGTGG AACCCAAGTG GCTTTGTAAA
4981 ATCATGGCAC AGATTTTGAC AGTGAAAGTG GAAGGTTGTC CAAAACACCC TAAGGGAATT
5041 ATTTCGCGTA GAGATGTGGA AAAATTTCTT TCAAAGAAAA GGAAATTTCC AAAGAACTAC
5101 ATGTCACAGT ATTTTAAGCT CCTAGAAAAA TTCCAGATTG CTTTGCCAAT AGGAGAAGAA
5161 TATTTGCTGG TTCCAAGCAG TTTGTCTGAC CACAGGCCTG TGATAGAGCT TCCCCATTGT
5221 GAGAACTCTG AAATTATCAT CCGACTATAT GAAATGCCTT ATTTTCCAAT GGGATTTTGG

5281 TCAAGATTAA TCAATCGATT ACTTGAGATT TCACCTTACA TGCTTTCAGG GAGAGAACGA
5341 GCACTTCGCC CAAACAGAAT GTATTGGCGA CAAGGCATTT ACTTAAATTG GTCTCCTGAA
5401 GCTTATTGTC TGGTAGGATC TGAAGTCTTA GACAATCATC CAGAGAGTTT CTTAAAAATT
5461 ACAGTTCCTT CTTGTAGAAA AGGCTGTATT CTTTTGGGCC AAGTTGTGGA CCACATTGAT
5521 TCTCTCATGG AAGAATGGTT TCCTGGGTTG CTGGAGATTG ATATTTGTGG TGAAGGAGAA
5581 ACTCTGTTGA AGAAATGGGC ATTATATAGT TTTAATGATG GCGAAGAACA TCAAAAAATC
5641 TTACTTGATG ACTTGATGAA GAAAGCAGAG GAAGGAGATC TCTTAGTAAA TCCAGATCAA
5701 CCAAGGCTCA CCATTCCAAT ATCTCAGATT GCCCCTGACT TGATTTTGGC TGACCTGCCT
5761 AGAAATATTA TGTTGAATAA TGATGAGTTG GAATTTGAAC AAGCTCCAGA GTTTCTCCTA
5821 GGTGATGGCA GTTTTGGATC AGTTTACCGA GCAGCCTATG AAGGAGAAGA AGTGGCTGTG
5881 AAGATTTTTA ATAAACATAC ATCACTCAGG CTGTTAAGAC AAGAGCTTGT GGTGCTTTGC
5941 CACCTCCACC ACCCCAGTTT GATATCTTTG CTGGCAGCTG GGATTCGTCC CCGGATGTTG
6001 GTGATGGAGT TAGCCTCCAA GGGTTCCTTG GATCGCCTGC TTCAGCAGGA CAAAGCCAGC
6061 CTCACTAGAA CCCTACAGCA CAGGATTGCA CTCCACGTAG CTGATGGTTT GAGATACCTC
6121 CACTCAGCCA TGATTATATA CCGAGACCTG AAACCCCACA ATGTGCTGCT TTTCACACTG
6181 TATCCCAATG CTGCCATCAT TGCAAAGATT GCTGACTACA GCATTGCTCA GTACTGCTGT
6241 AGAATGGGGA TAAAAACATC AGAGGGCACA CCAGGGTTTC GTGCACCTGA AGTTGCCAGA
6301 GGAAATGTCA TTTATAACCA ACAGGCTGAT GTTTATTCAT TTGGTTTACT ACTCTATGAC
6361 ATTTTGACAA CTGGAGGTAG AATAGTAGAG GGTTTGAAGT TTCCAAATGA GTTTGATGAA
6421 TTAGAAATAC AAGGAAAATT ACCTGATCCA GTTAAAGAAT ATGGTTGTGC CCCATGGCCT
6481 ATGGTTGAAA AATTAATTAA ACAGTGTTTG AAAGAAAATC CTCAAGAAAG GCCTACTTCT
6541 GCCCAGGTCT TTGACATTTT GAATTCAGCT GAATTAGTCT GTCTGACGAG ACGCATTTTA
6601 TTACCTAAAA ACGTAATTGT TGAATGCATG GTTGCTACAC ATCACAACAG CAGGAATGCA
6661 AGCATTTGGC TGGGCTGTGG GCACACCGAC AGAGGACAGC TCTCATTTCT TGACTTAAAT
6721 ACTGAAGGAT ACACTTCTGA GGAAGTTGCT GATAGTAGAA TATTGTGCTT AGCCTTGGTG
6781 CATCTTCCTG TTGAAAAGGA AAGCTGGATT GTGTCTGGGA CACAGTCTGG TACTCTCCTG
6841 GTCATCAATA CCGAAGATGG GAAAAAGAGA CATACCCTAG AAAAGATGAC TGATTCTGTC
6901 ACTTGTTTGT ATTGCAATTC CTTTTCCAAG CAAAGCAAAC AAAAAAATTT TCTTTTGGTT
6961 GGAACCGCTG ATGGCAAGTT AGCAATTTTT GAAGATAAGA CTGTTAAGCT TAAAGGAGCT
7021 GCTCCTTTGA AGATACTAAA TATAGGAAAT GTCAGTACTC CATTGATGTG TTTGAGTGAA
7081 TCCACAAATT CAACGGAAAG AAATGTAATG TGGGGAGGAT GTGGCACAAA GATTTTCTCC
7141 TTTTCTAATG ATTTCACCAT TCAGAAACTC ATTGAGACAA GAACAAGCCA ACTGTTTTCT
7201 TATGCAGCTT TCAGTGATTC CAACATCATA ACAGTGGTGG TAGACACTGC TCTCTATATT
7261 GCTAAGCAAA ATAGCCCTGT TGTGGAAGTG TGGGATAAGA AAACTGAAAA ACTCTGTGGA
7321 CTAATAGACT GCGTGCACTT TTTAAGGGAG GTAACGGTAA AAGAAAACAA GGAATCAAAA
7381 CACAAAATGT CTTATTCTGG GAGAGTGAAA ACCCTCTGCC TTCAGAAGAA CACTGCTCTT
7441 TGGATAGGAA CTGGAGGAGG CCATATTTTA CTCCTGGATC TTTCAACTCG TCGACTTATA
7501 CGTGTAATTT ACAACTTTTG TAATTCGGTC AGAGTCATGA TGACAGCACA GCTAGGAAGC
7561 CTTAAAAATG TCATGCTGGT ATTGGGCTAC AACCGGAAAA ATACTGAAGG TACACAAAAG
7621 CAGAAAGAGA TACAATCTTG CTTGACCGTT TGGGACATCA ATCTTCCACA TGAAGTGCAA
7681 AATTTAGAAA AACACATTGA AGTGAGAAAA GAATTAGCTG AAAAAATGAG ACGAACATCT
7741 GTTGAGTAA

Supplementary Sequence 6 | Reference sequence and editing site of the murine MECP2 W104X – eGFP cDNA construct on plasmid pTS1088 (Supplementary Note 2). Target adenosine is highlighted in red, linker is highlighted pink, eGFP sequence is highlighted green.

1 ATGGCCGCCG CTGCCGCCAC CGCCGCCGCC GCCGCCGCGC CGAGCGGAGG AGGAGGAGGA
61 GGCGAGGAGG AGAGACTGGA GGAAAAGTCA GAAGACCAGG ATCTCCAGGG CCTCAGAGAC
121 AAGCCACTGA AGTTTAAGAA GGCGAAGAAA GACAAGAAGG AGGACAAAGA AGGCAAGCAT
181 GAGCCACTAC AACCTTCAGC CCACCATTCT GCAGAGCCAG CAGAGGCAGG CAAAGCAGAA
241 ACATCAGAAA GCTCAGGCTC TGCCCCAGCA GTGCCAGAAG CCTCGGCTTC CCCCAAACAG
301 CGGCGCTCCA TTATCCGTGA CCGGGGACCT ATGTATGATG ACCCCACCTT GCCTGAAGGT
361 TAGACACGAA AGCTTAAACA AAGGAAGTCT GGCCGATCTG CTGGAAAGTA TGATGTATAT
421 TTGATCAATC CCCAGGGAAA AGCTTTTCGC TCTAAAGTAG AATTGATTGC ATACTTTGAA

481 AAGGTGGGAG ACACCTCCTT GGACCCTAAT GATTTTGACT TCACGGTAAC TGGGAGAGGG
541 AGCCCCTCCA GGAGAGAGCA GAAACCACCT AAGAAGCCCA AATCTCCCAA AGCTCCAGGA
601 ACTGGCAGGG GTCGGGGACG CCCCAAAGGG AGCGGCACTG GGAGACCAAA GGCAGCAGCA
661 TCAGAAGGTG TTCAGGTGAA AAGGGTCCTG GAGAAGAGCC CTGGGAAACT TGTTGTCAAG
721 ATGCCTTTCC AAGCATCGCC TGGGGGTAAG GGTGAGGGAG GTGGGGCTAC CACATCTGCC
781 CAGGTCATGG TGATCAAACG CCCTGGCAGA AAGCGAAAAG CTGAAGCTGA CCCCCAGGCC
841 ATTCCTAAGA AACGGGGTAG AAAGCCTGGG AGTGTGGTGG CAGCTGCTGC AGCTGAGGCC
901 AAAAAGAAAG CCGTGAAGGA GTCTTCCATA CGGTCTGTGC ATGAGACTGT GCTCCCCATC
961 AAGAAGCGCA AGACCCGGGA GACGGTCAGC ATCGAGGTCA AGGAAGTGGT GAAGCCCCTG
1021 CTGGTGTCCA CCCTTGGTGA GAAAAGCGGG AAGGGACTGA AGACCTGCAA GAGCCCTGGG
1081 CGTAAAAGCA AGGAGAGCAG CCCCAAGGGG CGCAGCAGCA GTGCCTCCTC CCCACCTAAG
1141 AAGGAGCACC ATCATCACCA CCATCACTCA GAGTCCACAA AGGCCCCCAT GCCACTGCTC
1201 CCATCCCCAC CCCCACCTGA GCCTGAGAGC TCTGAGGACC CCATCAGCCC CCCTGAGCCT
1261 CAGGACTTGA GCAGCAGCAT CTGCAAAGAA GAGAAGATGC CCCGAGGAGG CTCACTGGAA
1321 AGCGATGGCT GCCCCAAGGA GCCAGCTAAG ACTCAGCCTA TGGTCGCCAC CACTACCACA
1381 GTTGCAGAAA AGTACAAACA CCGAGGGGAG GGAGAGCGCA AAGACATTGT TTCATCTTCC
1441 ATGCCAAGGC CAAACAGAGA GGAGCCTGTG GACAGCCGGA CGCCCGTGAC CGAGAGAGTT
1501 AGCGGTACCG CGGGCCCGGG GTCCATCGCC ACCATGGTGA GCAAGGGCGA GGAGCTGTTC
1561 ACCGGGGTGG TGCCCATCCT GGTCGAGCTG GACGGCGACG TAAACGGCCA CAAGTTCAGC
1621 GTGTCCGGCG AGGGCGAGGG CGATGCCACC TACGGCAAGC TGACCCTGAA GTTCATCTGC
1681 ACCACCGGCA AGCTGCCCGT GCCCTGGCCC ACCCTCGTGA CCACCCTGAC CTACGGCGTG
1741 CAGTGCTTCA GCCGCTACCC CGACCACATG AAGCAGCACG ACTTCTTCAA GTCCGCCATG
1801 CCCGAAGGCT ACGTCCAGGA GCGCACCATC TTCTTCAAGG ACGACGGCAA CTACAAGACC
1861 CGCGCCGAGG TGAAGTTCGA GGGCGACACC CTGGTGAACC GCATCGAGCT GAAGGGCATC
1921 GACTTCAAGG AGGACGGCAA CATCCTGGGG CACAAGCTGG AGTACAACTA CAACAGCCAC
1981 AACGTCTATA TCATGGCCGA CAAGCAGAAG AACGGCATCA AGGTGAACTT CAAGATCCGC
2041 CACAACATCG AGGACGGCAG CGTGCAGCTC GCCGACCACT ACCAGCAGAA CACCCCCATC
2101 GGCGACGGCC CCGTGCTGCT GCCCGACAAC CACTACCTGA GCACCCAGTC CGCCCTGAGC
2161 AAAGACCCCA ACGAGAAGCG CGATCACATG GTCCTGCTGG AGTTCGTGAC CGCCGCCGGG
2221 ATCACTCTCG GCATGGACGA GCTGTACAAG TAA

References

1. Doherty, E.E. et al. Rational Design of RNA Editing Guide Strands: Cytidine Analogs at the Orphan Position. *Journal of the American Chemical Society* (2021).
2. Brinkman, H.F., Jauregui Matos, V., Mendoza, H.G., Doherty, E.E. & Beal, P.A. Nucleoside analogs in ADAR guide strands targeting 5′-U sites. *RSC Chemical Biology* (2022).
3. Zuker, M. & Stiegler, P. Optimal computer folding of large RNA sequences using thermodynamics and auxiliary information. *Nucleic Acids Res* **9**, 133-148 (1981).
4. Vickers, T.A., Rahdar, M., Prakash, T.P. & Crooke, S.T. Kinetic and subcellular analysis of PS-ASO/protein interactions with P54nrb and RNase H1. *Nucleic Acids Res* **47**, 10865-10880 (2019).
5. Hanswillemenke, A. et al. Profiling the interactome of oligonucleotide drugs by proximity biotinylation. *Nature Chemical Biology* (2024).
6. Shen, W., Liang, X.-h., Sun, H. & Crooke, S.T. 2′-Fluoro-modified phosphorothioate oligonucleotide can cause rapid degradation of P54nrb and PSF. *Nucleic Acids Res* **43**, 4569-4578 (2015).
7. Merkle, T. et al. Precise RNA editing by recruiting endogenous ADARs with antisense oligonucleotides. *Nature Biotechnology* **37**, 133-138 (2019).
8. Lumb, J.H. et al. DDX6 Represses Aberrant Activation of Interferon-Stimulated Genes. *Cell Reports* **20**, 819-831 (2017).
9. Li, H. & Durbin, R. Fast and accurate long-read alignment with Burrows–Wheeler transform. *Bioinformatics* **26**, 589-595 (2010).
10. McKenna, A. et al. The Genome Analysis Toolkit: a MapReduce framework for analyzing next-generation DNA sequencing data. *Genome Res* **20**, 1297-1303 (2010).
11. Ramaswami, G. et al. Accurate identification of human Alu and non-Alu RNA editing sites. *Nature Methods* **9**, 579-581 (2012).
12. Ramaswami, G. et al. Identifying RNA editing sites using RNA sequencing data alone. *Nature Methods* **10**, 128-132 (2013).
13. Li, H. et al. The Sequence Alignment/Map format and SAMtools. *Bioinformatics* **25**, 2078-2079 (2009).
14. Kent, W.J. BLAT--the BLAST-like alignment tool. *Genome Res* **12**, 656-664 (2002).
15. Wang, K., Li, M. & Hakonarson, H. ANNOVAR: functional annotation of genetic variants from high-throughput sequencing data. *Nucleic Acids Res* **38**, e164-e164 (2010).
16. Love, M.I., Huber, W. & Anders, S. Moderated estimation of fold change and dispersion for RNA-seq data with DESeq2. *Genome Biol* **15**, 550 (2014).
17. Eden, E., Navon, R., Steinfeld, I., Lipson, D. & Yakhini, Z. GOrilla: a tool for discovery and visualization of enriched GO terms in ranked gene lists. *BMC Bioinformatics* **10**, 48 (2009).
18. Borchert, N. et al. Proteogenomics of Pristionchus pacificus reveals distinct proteome structure of nematode models. *Genome Res* **20**, 837-846 (2010).
19. Rappsilber, J., Mann, M. & Ishihama, Y. Protocol for micro-purification, enrichment, pre-fractionation and storage of peptides for proteomics using StageTips. *Nat Protoc* **2**, 1896-1906 (2007).
20. Ohana, R.F. et al. Deciphering the Cellular Targets of Bioactive Compounds Using a Chloroalkane Capture Tag. *ACS Chem Biol* **10**, 2316-2324 (2015).
21. Cox, J. & Mann, M. MaxQuant enables high peptide identification rates, individualized p.p.b.-range mass accuracies and proteome-wide protein quantification. *Nat Biotechnol* **26**, 1367-1372 (2008).
22. Cox, J. et al. Andromeda: a peptide search engine integrated into the MaxQuant environment. *J Proteome Res* **10**, 1794-1805 (2011).
23. Elias, J.E. & Gygi, S.P. Target-decoy search strategy for increased confidence in large-scale protein identifications by mass spectrometry. *Nat Methods* **4**, 207-214 (2007).

24. Tyanova, S. et al. The Perseus computational platform for comprehensive analysis of (prote)omics data. *Nat Methods* **13**, 731-740 (2016).
25. Monian, P. et al. Endogenous ADAR-mediated RNA editing in non-human primates using stereopure chemically modified oligonucleotides. *Nature Biotechnology* (2022).
