# Supplementary material for: Stereo-random oligonucleotides enable efficient recruitment of ADAR in vitro and in vivo
Source: Nat Commun. 2025 Oct 3;16:8849. doi: 10.1038/s41467-025-64434-7 (PMC12494997; doi:10.1038/s41467-025-64434-7)
Supplement: Supplementary file 2 — Description of Additional Supplementary Files [file 41467_2025_64434_MOESM2_ESM.pdf]

Description of Additional Supplementary Files

File Name: Supplementary Data 1
Description: Supplementary Data 1 provides an overview of all nucleobase sequences, precise chemical modifications and respective names of each oligonucleotide used in the study. It contains the following columns:

- Target site: brief description of which (human or murine) transcript was targeted with the oligonucleotide, and nearest neighbor context of the target adenosine (in brackets).
- ON version: the name given to the referenced oligonucleotide in the study.
- Length: precise length of the oligonucleotide.
- ON symmetry: the length of each oligonucleotide terminus in relation to the orphan base (the nucleobase opposite of the target adenosine), in 5’ -> 3’ orientation.
- Sequence (5’ -> 3’): the precise nucleobase sequence and chemical modifications of each oligonucleotide. The legend of the chemical modifications is also depicted in the header.
