# Supplementary material for: Stereo-random oligonucleotides enable efficient recruitment of ADAR in vitro and in vivo
Source: Nat Commun. 2025 Oct 3;16:8849. doi: 10.1038/s41467-025-64434-7 (PMC12494997; doi:10.1038/s41467-025-64434-7)
Supplement: Supplementary file 4 — Reporting Summary [file 41467_2025_64434_MOESM4_ESM.pdf]

nature portfolio

Corresponding author(s): Thorsten Stafforst

Last updated by author(s): Jul 25, 2025

Reporting Summary

Nature Portfolio wishes to improve the reproducibility of the work that we publish. This form provides structure for consistency and transparency in reporting. For further information on Nature Portfolio policies, see our Editorial Policies and the Editorial Policy Checklist.

Statistics

For all statistical analyses, confirm that the following items are present in the figure legend, table legend, main text, or Methods section.

n/a	Confirmed	
☐	☒	The exact sample size (*n*) for each experimental group/condition, given as a discrete number and unit of measurement
☐	☒	A statement on whether measurements were taken from distinct samples or whether the same sample was measured repeatedly
☐	☒	The statistical test(s) used AND whether they are one- or two-sided<br>*Only common tests should be described solely by name; describe more complex techniques in the Methods section.*
☒	☐	A description of all covariates tested
☒	☐	A description of any assumptions or corrections, such as tests of normality and adjustment for multiple comparisons
☐	☒	A full description of the statistical parameters including central tendency (e.g. means) or other basic estimates (e.g. regression coefficient) AND variation (e.g. standard deviation) or associated estimates of uncertainty (e.g. confidence intervals)
☐	☒	For null hypothesis testing, the test statistic (e.g. *F*, *t*, *r*) with confidence intervals, effect sizes, degrees of freedom and *P* value noted<br>*Give P values as exact values whenever suitable.*
☒	☐	For Bayesian analysis, information on the choice of priors and Markov chain Monte Carlo settings
☒	☐	For hierarchical and complex designs, identification of the appropriate level for tests and full reporting of outcomes
☒	☐	Estimates of effect sizes (e.g. Cohen's *d*, Pearson's *r*), indicating how they were calculated

Our web collection on statistics for biologists contains articles on many of the points above.

Software and code

Policy information about availability of computer code

Data collection	No custom-made code was required to collect data.<br>For the RNA seq data, Next-generation sequencing of Poly(A)+ mRNA was done by CeGaT (Germany). The library was prepared with the TruSeq Stranded mRNA Library Prep Kit (Illumina, USA), and sequenced with the NovaSeq 6000 (50 M reads, 2 × 100 bp paired end, Illumina, USA).
Data analysis	Data were analyzed using Excel 2016 and GraphPad Prism 8 or 10, Figures were created with CorelDraw 2017, the manuscript was written with Word 2016.<br>Nikon NIS Offline Deconvolution 4.51 (deconvolution of microscopy images)<br>Image J win64 (adjustment of brightness and contrast of Western blots and denaturing urea PAGE gels, cropping of Western blots, performing maximum projection of deconvoluted microscopy images and assigning LUTs)<br>MaxQuant software package version 1.6.14.0 (processing of SILAC-MS/MS data)<br>Perseus software v1.6.15.0 (preparation of scatterplots)<br><br>RNA seq data (as previously described in Merkle et al., 2019):<br>Mapping of RNA-seq and reads: BWA (version 0.7.10) was used to align the reads to a combination of the reference genome sequences (hg19) and exonic sequences surrounding known splicing junctions from known gene models. Gene models were obtained through the UCSC Genome Browser for Gencode, RefSeq, Ensembl, and UCSC Genes. Unique and non-duplicate reads were subjected to local realignment and base score recalibration using the IndelRealigner and TableRecalibration from the Genome Analysis Toolkit (GATK, version 3.6).<br>Identification of editing sites from RNA-seq data: UnifiedGenotyper from GATK27 called variants from the mapped RNA-seq reads. In contrast to the usual practice of variant calling, the variants were identified with relatively loose criteria by using the UnifiedGenotyper tool. First, alll known human SNPs present in dbSNP, build 137 (except SNPs of molecular type “cDNA”; database version 135; http://www.ncbi.nlm.nih.gov/

SNP/), the 1000 Genomes Project, and the University of Washington Exome Sequencing Project (http://evs.gs.washington.edu/EVS/) were removed. Finally, variants were annotated using ANNOVAR (version 11122013) based on gene models from Gencode, RefSeq, Ensembl, and UCSC. The resulting sets of sites identified from RNA-seq data were compared with all sites available in the RADAR database and were subsequently referred to as 'known' sites if also found in RADAR, or 'novel' sites if not found. Code can also be downloaded at: http://lilab.stanford.edu/SNPiR/

For manuscripts utilizing custom algorithms or software that are central to the research but not yet described in published literature, software must be made available to editors and reviewers. We strongly encourage code deposition in a community repository (e.g. GitHub). See the Nature Portfolio guidelines for submitting code & software for further information.

Data

Policy information about availability of data

All manuscripts must include a data availability statement. This statement should provide the following information, where applicable:

- Accession codes, unique identifiers, or web links for publicly available datasets
- A description of any restrictions on data availability
- For clinical datasets or third party data, please ensure that the statement adheres to our policy

The datasets generated during and/or analyzed during the current study are available from the corresponding author on reasonable request.

Research involving human participants, their data, or biological material

Policy information about studies with human participants or human data. See also policy information about sex, gender (identity/presentation), and sexual orientation and race, ethnicity and racism.

Reporting on sex and gender: No research involving human participants.

Reporting on race, ethnicity, or other socially relevant groupings: No research involving human participants.

Population characteristics: No research involving human participants.

Recruitment: No research involving human participants.

Ethics oversight: No research involving human participants.

Note that full information on the approval of the study protocol must also be provided in the manuscript.

Field-specific reporting

Please select the one below that is the best fit for your research. If you are not sure, read the appropriate sections before making your selection.

☒ Life sciences ☐ Behavioural & social sciences ☐ Ecological, evolutionary & environmental sciences

For a reference copy of the document with all sections, see nature.com/documents/nr-reporting-summary-flat.pdf

Life sciences study design

All studies must disclose on these points even when the disclosure is negative.

Sample size: Experiments for evaluating editing yields via Sanger sequencing were mostly done in n=2-3 biological replicates in rare cases in n < 3 (max. n = 17). When possible, data points are displayed individually in the column or bar graphs. For the in vitro A1AT ELISA, each biological replicate was measured in technical triplicates.
The evaluation of the editing yields and wildtype A1AT serum levels for the animal experiment was done with n=2-5 mice per group. Each editing yield data point was measured individually as one technical replicate containing samples from multiple different liver lobes. To obtain the correlation of in vivo editing yield and A1AT serum level, samples from the same animal were used.
No sample size calculation was performed. The group sizes for cell culture and animal experiments were selected based on the prior knowledge of variation.
SILAC-MS/MS experiments were performed in duplicates with swapped SILAC labels. All protein groups identified and enrichment in bloth replicates can be found in the provided primary data.
RNA seq analysis was performed with two independent replicates per sample; the required sequencing depth was determined in a previous study (Vogel et al. Nature Methods 2018).

Data exclusions: No data was excluded.

Replication: All experiments could be reproduced, as shown in the manuscript, the number of replications and nature of replicates is always given in the figure caption.
As usual in the field of proteomics and due to complexity of SILAC experiments, two replicates were performed and correlation was checked. The RNA seq data was gathered from two biological replicates.

Randomization	All samples were treated according to the same protocols side-by-side with the respective controls and thus, there was no requirement for randomization.
Blinding	Blinding was performed during the downstream analysis of SILAC-MS/MS experiments (sample preparation and measurement). For all other experiments, no blinding was performed due to the involvement of several experimentators.

Reporting for specific materials, systems and methods

We require information from authors about some types of materials, experimental systems and methods used in many studies. Here, indicate whether each material, system or method listed is relevant to your study. If you are not sure if a list item applies to your research, read the appropriate section before selecting a response.

Materials & experimental systems

n/a	Involved in the study
☐	☒ Antibodies
☐	☒ Eukaryotic cell lines
☒	☐ Palaeontology and archaeology
☐	☒ Animals and other organisms
☒	☐ Clinical data
☒	☐ Dual use research of concern
☒	☐ Plants

Methods

n/a	Involved in the study
☒	☐ ChIP-seq
☒	☐ Flow cytometry
☒	☐ MRI-based neuroimaging

Antibodies

Antibodies used	Polyclonal rabbit anti-human AlAT (cat. no. A0012, Agilent Technologies, lot no. 20079116, used as 1:500 dilution); polyclonal goat anti-human A1AT-HRP conjugated antibody (Bethyl Laboratories, cat. no. A80-122P, lot no. 27, used as 1:10 000 dilution); monoclonal mouse anti-human wildtype-A1AT antibody (purchased from Mark Brantly lab, University of Florida via MTA, used as 0.167 µg/ml solution); monoclonal mouse anti-human NONO antibody (Santa Cruz Biotechnology, no. sc-376865, used as 1:1000 dilution); monoclonal mouse anti-human GAPDH antibody (ThermoFisher Scientific, no. MA5-15738, used as 1:1000 dilution); polyclonal goat anti-mouse lgG-HRP conjugated antibody (Jackson Immunoresearch Laboratories, no. 115-035-003, used as 1:10 000 dilution)
Validation	Polyclonal rabbit anti-human A1AT:<br>PMID: 7008496<br>PMID: 3873300<br>PMID: 3518416<br><br>Polyclonal goat anti-human A1AT-HRP conjugated antibody:<br>PMID: 35121111<br>PMID: 33117614<br>PMID: 30840913<br>PMID: 23926210<br>PMID: 19073710<br><br>Monoclonal mouse anti-human wildtype-A1AT antibody:<br>PMID: 23195820<br>PMID: 31723592<br><br>Monoclonal mouse anti-human NONO antibody:<br>PMID: 25855809<br>PMID: 35852833<br><br>Monoclonal mouse anti-human GAPDH antibody:<br>PMID: 31519936

Eukaryotic cell lines

Policy information about cell lines and Sex and Gender in Research

Cell line source(s)	HeLa: ATCC (Cat.No.: ATCC CCL-2), U2OS: kind donation from Prof. Elmar Schiebel, SK-N-BE(2): ATCC (Cat.No.: ATCC CRL-2271), Huh7:CLS (CLS GmbH, Heidelberg, Cat.No.: 300156), HepG2: DSMZ (DSMZ, Braunschweig, Germany Cat.No.: ACC180), A549: ECACC (European Collection of Authenticated Cell Cultures ECACC 86012804), SH-SY5Y: ATCC (Cat.No.: ATCC CRL-2266), HEK-Flp-In T-Rex-A1p110 (R78007, Thermo Fisher scientific, stably transfected with ADAR1 p110 vector in our lab), HEK-Flp-In T-Rex-A1p150 (R78007, Thermo Fisher scientific, stably transfected with ADAR1p150 vector in our lab), HEK-Flp-In T-Rex-ADAR2 (R78007, Thermo Fisher scientific, stably transfected with ADAR2 vector in our lab), Normal Human Astrocytes: Lonza (NHA, Cat.No.: CC-2565), Human Retinal Pigment Epithelial Cells: Lonza (H-RPE, Cat.No.: 194987), Normal

	Human Bronchial Epithelial Cells: Lonza (NHBE, Cat.No.: CC-2540) and Primary Human Hepatocytes: Lonza (PHH,Cat.No.: HUCPI, lot no. HUM182041)
Authentication	Authentication via STR profiling was performed by the commercial suppliers before purchase of the material. Cell lines were not additionally authenticated by us.
Mycoplasma contamination	NHA, RPE, and NHBE were certified as mycoplasma-free by the supplier. All other cell lines have been tested as mycoplasma-free in house.
Commonly misidentified lines (See ICLAC register)	None were used.

Animals and other research organisms

Policy information about studies involving animals; ARRIVE guidelines recommended for reporting animal research, and Sex and Gender in Research

Laboratory animals	Species: mouse, strain: C57BL/6J, disease: alpha-1-antitrypsin deficiency caused by genomic integration of human SERPINA1 E342K transgene (PMID 2784798). Mice were housed on a 12:12 light-dark cycle, with ad libitum access to food and water at 21°C/52% humidity. All groups consisted of SERPINA1 E342K-homozygous male and female mice.<br>The animals were between 8 and 10 weeks old at the time of oligonucleotide treatment.
Wild animals	No wild animals were used in this study.
Reporting on sex	Not collected.
Field-collected samples	No field-collected samples were used in this study.
Ethics oversight	All animal procedures were approved by the Administrative Panel on Laboratory Animal Care (APLAC) of Stanford University.

Note that full information on the approval of the study protocol must also be provided in the manuscript.
